# Taurine from tumour niche drives glycolysis to promote leukaemogenesis

**DOI:** 10.1038/s41586-025-09018-7

**Published:** 2025-05-14

**Authors:** Sonali Sharma, Benjamin J. Rodems, Cameron D. Baker, Christina M. Kaszuba, Edgardo I. Franco, Bradley R. Smith, Takashi Ito, Kyle Swovick, Kevin Welle, Yi Zhang, Philip Rock, Francisco A. Chaves, Sina Ghaemmaghami, Laura M. Calvi, Archan Ganguly, W. Richard Burack, Michael W. Becker, Jane L. Liesveld, Paul S. Brookes, Joshua C. Munger, Craig T. Jordan, John M. Ashton, Jeevisha Bajaj

**Affiliations:** 1 Department of Biomedical Genetics, University of Rochester Medical Center, Rochester, NY USA; 2 Wilmot Cancer Institute, University of Rochester Medical Center, Rochester, NY USA; 3 Genomics Research Center, University of Rochester Medical Center, Rochester, NY USA; 4 Department of Biomedical Engineering, University of Rochester, Rochester, NY USA; 5 Department of Biochemistry and Biophysics, University of Rochester Medical Center, Rochester, NY USA; 6 Department of Bioscience and Technology, Graduate School of Bioscience and Technology, Fukui Prefectural University, Fukui, Japan; 7 Mass Spectrometry Resource Laboratory, University of Rochester, Rochester, NY USA; 8 Department of Pathology and Laboratory Medicine, University of Rochester Medical Center, Rochester, NY USA; 9 Department of Biology, University of Rochester, Rochester, NY USA; 10 Department of Medicine, University of Rochester Medical Center, Rochester, NY USA; 11 Department of Neuroscience, University of Rochester Medical Center, Rochester, NY USA; 12 Department of Anesthesiology, University of Rochester Medical Center, Rochester, NY USA; 13 Division of Hematology, Department of Medicine, University of Colorado Anschutz Medical Campus, Aurora, CO USA

**Keywords:** Cancer, Cancer microenvironment, Cancer metabolism

## Abstract

Signals from the microenvironment are known to be critical for development, stem cell self-renewal and oncogenic progression. Although some niche-driven signals that promote cancer progression have been identified^1–5^, concerted efforts to map disease-relevant microenvironmental ligands of cancer stem cell receptors have been lacking. Here, we use temporal single-cell RNA-sequencing (scRNA-seq) to identify molecular cues from the bone marrow stromal niche that engage leukaemia stem-enriched cells (LSCs) during oncogenic progression. We integrate these data with our human LSC RNA-seq and in vivo CRISPR screen of LSC dependencies^6^ to identify LSC–niche interactions that are essential for leukaemogenesis. These analyses identify the taurine–taurine transporter (TAUT) axis as a critical dependency of aggressive myeloid leukaemias. We find that cysteine dioxygenase type 1 (CDO1)-driven taurine biosynthesis is restricted to osteolineage cells, and increases during myeloid disease progression. Blocking CDO1 expression in osteolineage cells impairs LSC growth and improves survival outcomes. Using TAUT genetic loss-of-function mouse models and patient-derived acute myeloid leukaemia (AML) cells, we show that TAUT inhibition significantly impairs in vivo myeloid leukaemia progression. Consistent with elevated TAUT expression in venetoclax-resistant AML, TAUT inhibition synergizes with venetoclax to block the growth of primary human AML cells. Mechanistically, our multiomic approaches indicate that the loss of taurine uptake inhibits RAG-GTP dependent mTOR activation and downstream glycolysis. Collectively, our work establishes the temporal landscape of stromal signals during leukaemia progression and identifies taurine as a key regulator of myeloid malignancies.

## Abstract (web summary)

The taurine–taurine transporter axis is a critical dependency of aggressive myeloid leukaemias.

## Main

Signals from the tumour microenvironment (TME) can regulate initiation, progression and immune evasion of tumours^1–5,7–10^. While scRNA-seq analysis has identified cellular TME components, especially in solid tumours^11,12^, concerted efforts to link ligands from the changing TME landscape with cognate receptors on cancer cells have been lacking. As cell surface proteins are particularly amenable to therapeutic targeting, functional characterization of their interactions with the TME are of considerable clinical interest.

Aggressive therapy-resistant myeloid leukaemias, such as blast-crisis-phase chronic myeloid leukaemia (bcCML) and AML, initiate and expand in a complex bone marrow microenvironment. Although previous studies have described the cellular composition of normal bone marrow niche^13–15^, their dynamic alterations during leukaemia progression remain undefined. We use scRNA-seq to establish temporal changes in bone marrow microenvironment populations, and in niche-driven signals during disease progression. To define TME ligands that are essential for leukaemogenesis, we focused on cognate cell surface receptors enriched in LSCs as compared to healthy controls, and those essential for in vivo leukaemia progression^6^. This approach identified signals that are known to be critical for cancer growth, such as KIT–KITL and CD47–thrombospondin 1^16^, as well as multiple new signalling axes.

Of these signals, TAUT, encoded by *SLC6A6*, was strongly associated with poor prognosis in human leukaemias and emerged as a key regulator of AML. As taurine can be neuroprotective, mitigate the side-effects of chemotherapy^17^ or support anti-cancer immunity^18^, a cancer-promoting role of taurine has not been considered. We examined if blocking taurine production in the TME impairs LSC function. We used genetic tools to establish whether TAUT expression in cancer cells controls the growth of aggressive myeloid leukaemias. Using metabolomic, proteomic and transcriptomic approaches, we identify downstream mechanisms by which taurine regulates leukaemia growth.

## Temporal changes in leukaemia niche

**Fig. 1:**
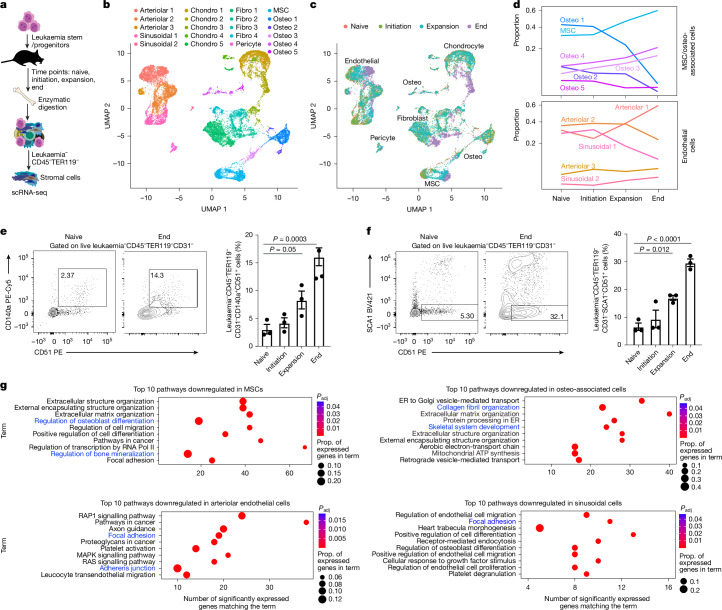
Temporal scRNA-seq analysis of myeloid leukaemia bone marrow microenvironment. **a**, The experimental strategy used to determine the impact of bcCML progression on bone-marrow microenvironment remodelling. **b**, A uniform manifold approximation and projection (UMAP) analysis of 15,695 non-haematopoietic cells from bone and bone marrow shows 21 distinct bone marrow stromal cell clusters (*n* = 3 (naive), n = 6 (initiation), n = 7 (expansion) and *n* = 9 (end) mice). The colour key indicates subclusters. Chrondro, chondrocyte; fibro, fibroblast; osteo, osteo-associated cell. **c**, UMAP plot of major population clusters over time (naive, 0 days; initiation, 2 and 4 days; expansion, 7 and 9 days; end, 11 and 14 days after transplant; the colours represent different stages of disease). **d**, The proportion (prop.) of MSCs/osteolineage cells (top) and endothelial cells (bottom) over time. **e**, Representative fluorescence-activated cell sorting (FACS) plots and quantification of MSC frequency over time. **f**, Representative FACS plots and quantification of the osteolineage cell frequency over time. For **e** and **f**, data are mean ± s.e.m. *n* = 3 animals per timepoint. Statistical analysis was performed using one-way analysis of variance (ANOVA). **g**, Unbiased Enrichr analysis showing the top 10 downregulated pathways by population cluster in MSCs, and osteo-associated, arteriolar and sinusoidal endothelial populations. Blue text represents pathways of interest. ER, endoplasmic reticulum. The mouse image in **a** is adapted from ref. ^6^, Springer Nature America. Source data

**Extended Data Fig. 1:**
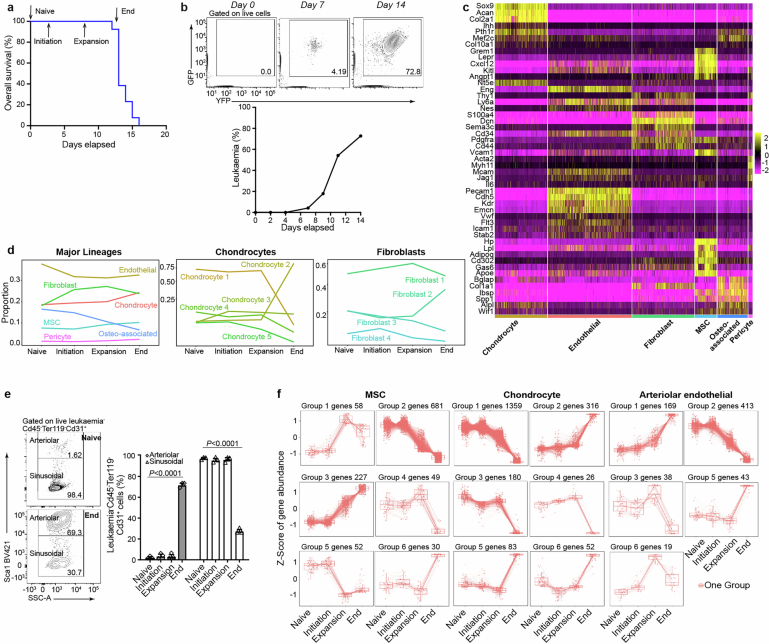
Temporal changes in leukaemia bone marrow microenvironment. **a**, Survival curve shows bcCML progression in unirradiated recipients, indicating time course for scRNA-seq experiment (naïve, initiation, expansion, and end). **b**, Representative FACS plots and graph show changes in leukaemia engraftment over time (2 or 3 mice pooled per timepoint). **c**, Heatmap of significantly expressed marker genes for non-haematopoietic Bone Marrow (BM) populations. **d**, Line graphs show temporal changes in the proportion of all major lineages, and in sub-clusters of chondrocyte and fibroblasts. **e**, Representative FACS plots and graph show changes in BM sinusoidal and arteriolar endothelial cell frequency over time (mean ± s.e.m.; n = 3 animals per timepoint, two-way ANOVA). **f**, Gene clusters associated with changes in MSC, chondrocyte, and arteriolar endothelial populations during disease progression. Source data

To define temporal changes in non-immune bone marrow microenvironment populations during myeloid leukaemia progression, we used bcCML LSCs, which can grow in unirradiated mouse recipients (Extended Data Fig. 1a,b). Bone marrow stromal populations (leukaemia^−^CD45^−^TER119^−^) were isolated from leukaemic cohorts at distinct stages of disease progression and analysed using scRNA-seq (Fig. 1a). Gene-expression-based clustering identified 21 lineage clusters with transitional subsets covering endothelial cells, chondrocytes, fibroblasts, pericytes, mesenchymal stromal cells (MSCs) and osteo-associated populations (Fig. 1b and Extended Data Fig. 1c,d) and showed significant remodelling with disease progression (Fig. 1c,d). Our flow-cytometry-based analyses of an independent cohort of leukaemic mice validated these changes, showing increases in MSCs, osteolineage and arteriolar endothelial cells, with a concomitant decline in sinusoidal endothelial populations (Fig. 1e,f and Extended Data Fig. 1e).

**Extended Data Fig. 2:**
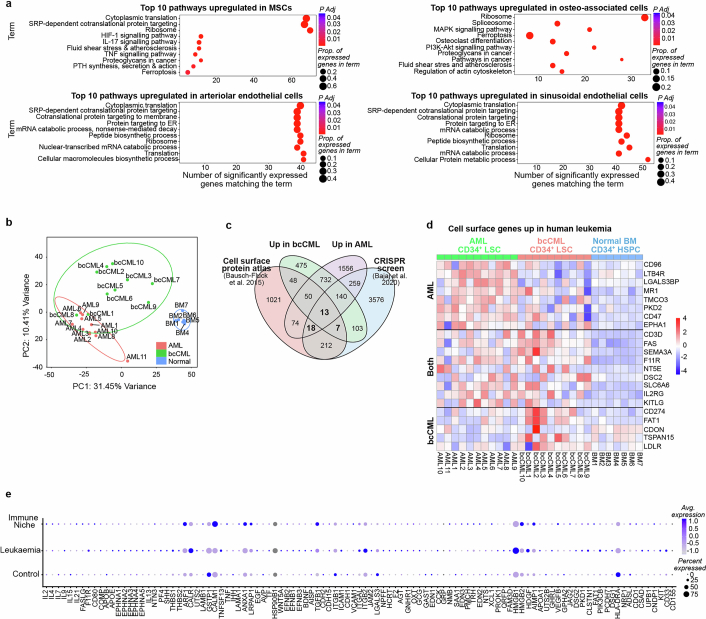
Signals from bone marrow niche interacting with human LSC cell surface receptors. **a**, Unbiased Enrichr analysis shows top 10 upregulated pathways by population cluster in MSCs, osteo-associated, arteriolar, and sinusoidal endothelial populations. **b**, PCA-plot shows the distribution of 7 CD34^+^ healthy donor BM HSPCs, 10 bcCML CD34^+^ LSCs, and 11 AML CD34^+^ LSCs from human samples. **c**, Overlap between genes upregulated in bcCML and AML CD34^+^ cells compared to normal CD34^+^ cells, proteins expressed on cell surface^19^, and those that drop-out by 2-fold or more in the leukaemia in vivo CRISPR screen^6^. **d**, Heatmap shows r-log normalized RNA expression of cell surface receptors upregulated in AML, bcCML or both. **e**, Average expression of ligands for cell surface proteins in primary human adult and paediatric AML cells, human AML immune microenvironment populations, and in normal cells^20^.

Clustering analyses of genes expressed in major lineages identified gene sets with similar changes in expression (Extended Data Fig. 1f), suggesting that these may represent altered stromal cell fate. Pathway analysis showed that osteoblast differentiation and bone mineralization were downregulated in MSCs and osteo-associated cells, and focal adhesion and cell migration were downregulated in endothelial populations, indicating that MSC and endothelial function may be impaired with disease progression (Fig. 1g and Extended Data Fig. 2a). Collectively, our temporal analysis of the TME identifies dynamic changes in gene expression of stromal subpopulations during leukaemia progression.

## Ligand–receptor interactome

**Fig. 2:**
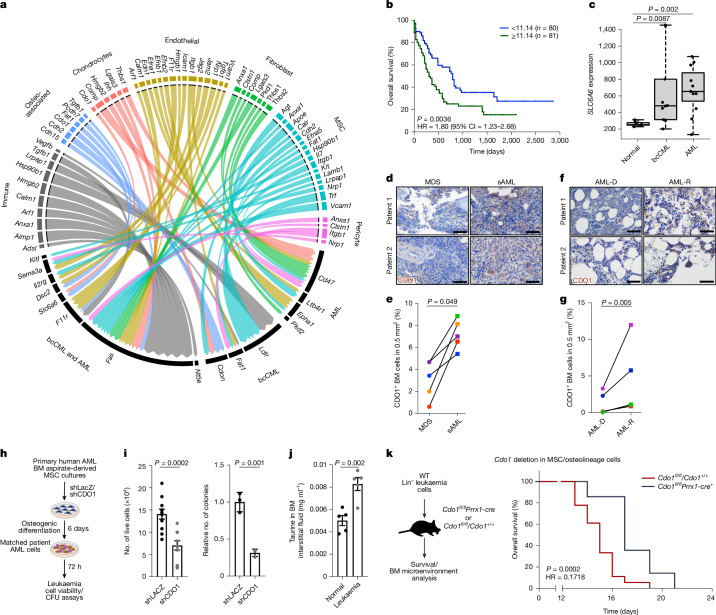
Bone marrow microenvironmental ligands for LSC-specific cell surface receptors. **a**, Circos plot showing leukaemia cell surface receptors and cognate stromal-cell-derived ligands. **b**, Kaplan–Meier curves of human patients with leukaemia with low (<11.14, *n* = 80) or high (≥11.14, *n* = 81) *SLC6A6* expression (TCGA-LAML; Xena Browser; log-rank test). CI, confidence interval; HR, hazard ratio. **c**, Normalized *SLC6A6* expression in CD43^+^ cells from normal human bone marrow (BM) samples and samples from patients with bcCML and AML. *n* = 7 (bone marrow), *n* = 10 (bcCML) and *n* = 11 (AML). For the box plots, the centre line shows the median, the box limits show the interquartile range and the whiskers represent the minimum and maximum values, respectively. Statistical analysis was performed using DESeq2-implemented Wald tests. **d**–**g**, Representative IHC images (**d**,**f**) and quantification (**e**,**g**) of CDO1 expression in matched patient bone marrow biopsies at MDS diagnosis and after AML transformation (**d**,**e**), or at AML diagnosis (AML-D) and relapse (AML-R) (**f**,**g**). *n* = 5 independent patients per cohort. Each colour represents a patient sample. Statistical analysis was performed using two-tailed ratio paired *t*-tests. **h**, The strategy used to determine the impact of inhibiting CDO1 in human bone marrow MSCs on co-cultured AML cells (MSCs and AML cells were derived from the same patient). CFU, colony-forming unit. **i**, The number of live LSCs (left; data are mean ± s.e.m.; *n* = 11 independent culture wells per cohort; data were combined from two independent experiments) and their colony-forming ability (right; data are mean ± s.d.; *n* = 3 independent culture wells per cohort) after coculture with AML MSCs. **j**, Taurine quantity per femur in control and leukaemic mice, as determined using colourimetric analysis, 12 days after transplant. Data are mean ± s.e.m. *n* = 5 animals per cohort. Data were combined from two independent experiments. For **i** and **j**, statistical analysis was performed using unpaired two-tailed Student’s *t*-tests. **k**, Experimental strategy and survival curve, showing the impact of blocking taurine production by MSC/osteolineage populations in vivo on bcCML progression in unirradiated recipients. *n* = 18 (*Cdo1*^*fl/fl*^/*Cdo1*^*+/+*^) and *n* = 14 (*Cdo1*^*fl/fl*^*Prrx1-cre*^*+*^). Data were combined from four independent experiments. Statistical analysis was performed using the log-rank test. WT, wild type. Scale bars, 50 µm (**d** and **f**). The mouse images in **h** and **k** are adapted from ref. ^6^, Springer Nature America. Source data

To define the functional relevance of microenvironmental changes on leukaemogenesis, we focused on proteins expressed on LSC cell surface that act as receptors for niche-driven signals. To identify LSC receptors associated with disease progression, we performed RNA-seq analysis of human AML and bcCML CD34^+^ cells, and healthy donor bone marrow CD34^+^ haematopoietic stem and progenitor cells (HSPCs; Extended Data Fig. 2b). We noted significant (adjusted *P* (*P*_adj_) < 0.05) upregulation of 1,569 genes in bcCML, 2,842 genes in AML and 2,331 genes in both bcCML and AML compared with normal HSPCs. To identify signals that are functionally relevant for disease progression, we found cell surface genes^19^ that drop out by twofold or more in our genome-wide in vivo leukaemia CRISPR screen^6^. This identified 13 cell surface signals common to both bcCML and AML, 18 unique to AML and 7 unique to bcCML. Of these 38 genes, 16 were misannotated as cell surface in the reference dataset^19^ and were removed from further analysis (Methods and Extended Data Fig. 2c,d). We used NicheNet and the published literature to identify ligands for these receptors, especially those that were significantly upregulated in our TME scRNA-seq data and in the human AML immune microenvironment^20^ (Extended Data Fig. 2e). This led to the exclusion of receptors with no known ligands (*MR1*, *TMCO3* and *TSPAN15*) as well as those of which the ligands were not significantly enriched in any TME population (*LGALS3BP*, *CD96*, *CD274* and *CD3D*). We therefore generated a unique map of TME ligands for 15 LSC receptors that are essential for disease progression (Fig. 2a).

**Extended Data Fig. 3:**
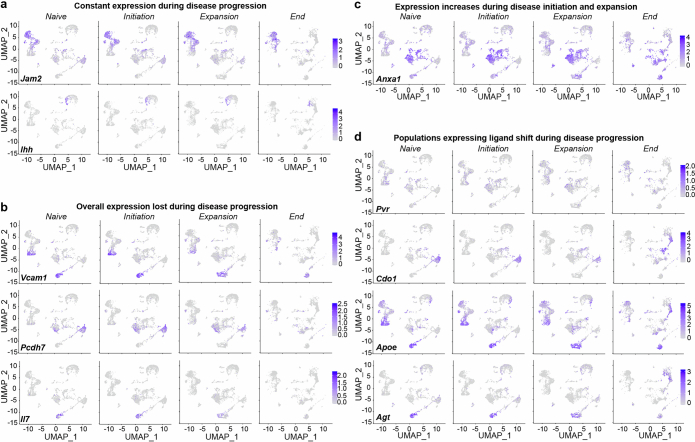
Temporal changes in niche-driven signals. **a**-**d**, UMAP plot of indicated gene expression in microenvironmental populations over time (naïve: 0 d, initiation: 2 and 4 days, expansion: 7 and 9 days and, end:11 and 14 days post-transplant).

We next determined whether disease progression could alter the expression of LSC-specific TME ligands, and identified four distinct patterns of ligand expression. While the expression of some genes remained steady, for example, *Jam2* in arteriolar endothelial cells and *Ihh* in chondrocytes, the expression of *Vcam1*, *Pcdh7* and *Il7* was lost in the TME, especially at the end point. Expression of *Anxa1* first increased but then declined towards the end. The fourth category of genes was always expressed, but the microenvironmental populations expressing them changed. These included *Pvr*, *Cdo1*, *Apoe* and *Agt* (Extended Data Fig. 3a–d). Our analysis capturing dynamic changes in the TME identifies signals that may be necessary not only at different stages of disease progression, but also those critical during the entire course of disease.

## TME signals support leukaemogenesis

**Extended Data Fig. 4:**
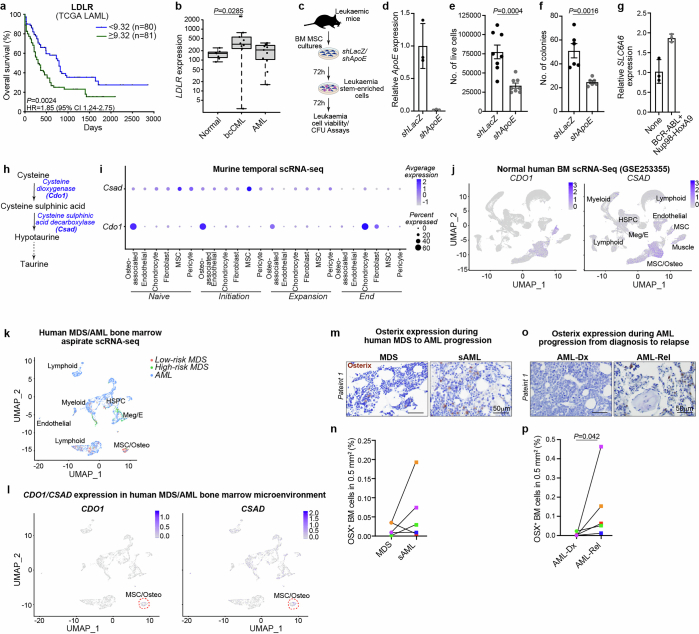
CDO1 expression in human leukaemia microenvironment. **a**, Kaplan-Meier curves of human leukaemia patients with high (<9.32, n = 80) or low (≥9.32, n = 81) LDLR expression (TCGA-LAML; Xena Browser; log-rank test). **b**, Normalized *LDLR* expression in CD34^+^ cells from human bcCML, AML, or normal BM (n = 7 normal BM, n = 10 bcCML, n = 11 AML; central line, box, and whiskers represent median, interquartile range or IQR, minimum/maximum within 1.5*IQR respectively; DESeq2 implemented Wald test). **c, d**, Experimental strategy (**c**) and relative *Apoe* expression (**d**) in MSCs transduced with shRNAs targeting *LacZ* or *Apoe* (mean ± s.d.; n = 3 technical replicates per cohort). **e,f**, Number of live leukaemia cells (**e**) and their colony forming ability (CFU) (**f**) post co-culture with MSCs (mean ± s.e.m.; **e**, n = 8 independent culture wells; **f**, n = 6 independent culture wells, data combined from two independent experiments; unpaired two-tailed Student’s t-test). **g**, Slc6a6 expression in KLS cells infected with BCR-ABL and NUP98-HOXA9 (BA-NH) oncogenes for 48 h (mean ± s.d.; n = 3 technical replicates per cohort). **h**, Taurine biosynthesis pathway. **i**, Dot plot of *Cdo1* and *Csad* expression during disease progression. **j**, UMAP plot of *CDO1* and *CSAD* expression in normal human BM^23^. **k**, UMAP plot of microenvironmental cells in three human MDS or AML BM aspirates. **l**, UMAP plot of *CDO1* and *CSAD* in human MDS and AML BM aspirates. **m**-**p**, Representative IHC images and quantification of Osterix expression in matched human BM biopsies at MDS diagnosis and AML transformation (**m, n**) or AML diagnosis and relapse (**o,p**) (n = 5 independent patients per cohort; each colour represents a patient sample; two-tailed ratio paired t-test). The mouse image in **c** is adapted from ref. ^6^, Springer Nature America. Source data

Multiple TME–LSC signalling axes identified in our interactome may have a functional role in leukaemia progression. However, we were interested in genes associated with unfavourable outcomes in human patients with AML. Of the 22 genes upregulated in human LSCs, only low-density lipoprotein receptor (*LDLR*) and *SLC6A6* were significantly associated with poor prognosis in AML (Fig. 2a–c and Extended Data Fig. 4a,b). We therefore tested the functional requirement of TME-derived ligands of LDLR and SLC6A6 on LSC growth. One of the primary LDLR ligands, apolipoprotein E (APOE)^21^, was highly expressed in MSCs (Extended Data Fig. 3d). To test the role of APOE from MSCs on LSC function, we co-cultured bcCML LSCs with MSCs transduced with short hairpin RNAs (shRNAs) targeting *Apoe* or *LacZ* (control; Extended Data Fig. 4c). Co-culture with MSCs lacking *Apoe* expression led to significant reduction in both the viability and colony-forming ability of LSCs (around 2.1-fold lower than the controls; Extended Data Fig. 4d–f).

**Extended Data Fig. 5:**
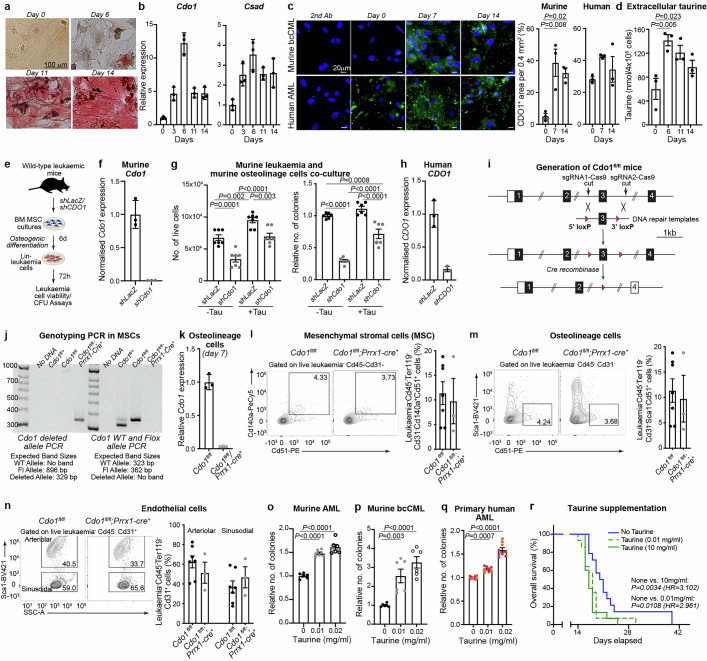
Inhibiting taurine synthesis in osteolineage cells impairs LSC growth. **a, b**, Alizarin red staining (**a**), and *Cdo1* and *Csad* expression (**b**) in MSCs undergoing osteogenic differentiation (mean ± s.d.; n = 3 technical replicates per cohort). **c**, CDO1 expression in murine leukaemia and patient AML MSCs undergoing osteogenic differentiation (mean ± s.e.m.; n = 3 replicates per time point). **d**, Taurine in MSC culture media during osteogenic differentiation (mean ± s.e.m.; n = 3 replicates; secreted over 48–72 h). **e,f**, Experimental strategy (**e**) and *Cdo1* expression (**f**) in MSCs transduced with shRNAs targeting *LacZ* or *Cdo1* (mean ± s.d.; n = 3 technical replicates per cohort). **g**, Live leukaemia cells (left), and CFU (right), post coculture with bcCML MSCs (mean ± s.e.m.; n = 7 independent culture wells per cohort (live); n = 6 (CFU); data combined from 2 independent experiments; one-way ANOVA). **h**, *CDO1* expression in 293 T transduced with shRNAs targeting *LACZ* or *CDO1* (mean ± s.d.; n = 3 technical replicates per cohort). **i**, *Cdo1*^*fl/fl*^ model. **j,k**, PCR of *Cdo1* (**j**) and *Cdo1* expression (**k**) in *Cdo1*^*fl/fl*^ and *Cdo1*^*fl/fl*^*;Prrx1-Cre*^*+*^ MSCs following osteogenic differentiation (mean ± s.d.; n = 3 technical replicates per cohort). **l**-**n**, BM stroma in leukaemic control and *Cdo1*^*fl/fl*^*;Prrx1-Cre*^*+*^ mice: MSCs (**l**), osteolineage cells (**m**), and, endothelial cells (**n**) (mean ± s.e.m.; n = 7 *Cdo1*^*fl/fl*^ and n = 3 *Cdo1*^*fl/fl*^*;Prrx1-Cre*^*+*^; data combined from three independent experiments; unpaired two-tailed Student’s t-test). **o-q**, CFU of murine cKit^+^ AML cells (**o**), murine Lin^−^ LSCs (**p**), or primary human AML cells (**q**) with taurine (mean ± s.e.m.; n = 3 independent culture wells from n = 2 murine AML, n = 2 murine bcCML, and n = 3 primary human AML samples; data combined from two independent experiments; one-way ANOVA). **r**, Impact of taurine supplements on murine bcCML progression in unirradiated recipients (n = 14 no taurine and 0.01 mg/ml taurine, n = 15 10 mg/ml taurine; data combined from three independent experiments; log-rank test). The mouse image in **e** is adapted from ref. ^6^, Springer Nature America. Source data

*SLC6A6* encodes TAUT, which has high affinity for the non-essential amino acid taurine, and low affinity for β-alanine (taurine *K*_m_ = 4.5 μM versus β-alanine *K*_m_ = 56 μM)^22^. Our experiments indicate that *Slc6a6* expression can be directly induced by oncogenes (Extended Data Fig. 4g). While the leukaemia TME did not express enzymes required for β-alanine synthesis (*Gadl1* and *Cndp1*), those needed for taurine biosynthesis were expressed in osteo-associated cells (*Cdo1* and *Csad*; Extended Data Fig. 4h,i). Our analysis of publicly available human bone marrow stromal cell scRNA-seq data using surgical samples from osteoarthritis patients^23^ showed that *CDO1* expression is restricted to MSC and osteolineage populations (Extended Data Fig. 4j). To test whether *CDO1* is expressed in the human leukaemia TME, we performed an scRNA-seq analysis of CD45^−^ stromal cells from three myelodysplastic syndrome (MDS) and AML bone marrow aspirates. Our analysis of the limited numbers of stromal cells in these samples indicate that *CDO1* expression is restricted to the MSC/osteolineage cells (Extended Data Fig. 4k,l), as we see in mouse samples. Notably, we found a marked increase in CDO1 protein expression in nearly all matched biopsies of patients who progressed from MDS to secondary AML, and those who relapsed after AML diagnosis (Fig. 2d–g and Extended Data Fig. 4m–p). While CDO1 protein was expressed in undifferentiated human AML MSCs, its expression increased during in vitro mouse and human AML MSC osteogenic differentiation (Extended Data Fig. 5a–c). Thus, CDO1 may be broadly expressed in immature MSCs as well as differentiating osteolineage cells in human bone marrow. Importantly, taurine levels in extracellular medium increased during MSC differentiation, indicating that elevated CDO1 expression is correlated with taurine production (Extended Data Fig. 5d).

To determine a functional role of taurine from osteolineage cells, we tested the impact of inhibiting taurine biosynthesis in mouse and human leukaemia bone-marrow-derived MSCs on co-cultured leukaemia cells. Our experiments showed that mouse LSCs co-cultured with osteolineage cells lacking *Cdo1* expression were significantly less viable (about 2-fold lower than the controls) and formed fewer colonies (3.4-fold less than controls), which could be rescued by supplementing cultures with taurine (Extended Data Fig. 5e–g). Similarly, inhibiting CDO1 in osteolineage cultures from MSCs derived from patients with AML impaired survival and colony-formation of co-cultured AML cells from the same patient by 2- to 3.2-fold (Fig. 2h,i and Extended Data Fig. 5h), indicating that taurine produced by osteolineage cells promotes LSC growth and survival.

Consistent with a functional role of taurine from the TME in leukaemia progression, taurine levels were 1.7-fold higher in leukaemic bone marrow interstitial fluid as compared to the controls (Fig. 2j). In mice, the majority of taurine is synthesized in the liver from cysteine^24^. To determine whether taurine produced locally in the bone marrow niche is essential for leukaemia growth, we generated a new *Cdo1*^*fl/fl*^ mouse model and crossed it to MSC/osteolineage-specific *Prrx1-cre* mice (Extended Data Fig. 5i–k). We used these as recipients for LSCs and monitored survival, and the impact on bone marrow microenvironment populations. Our experiments showed that *Cdo1*^*fl/fl*^*Prrx1-cre*^*+*^ mice lived around 13.5% longer than the controls, indicating that taurine produced locally in the leukaemia TME can support disease progression, at least in part (Fig. 2k). We did not find any significant changes in the composition of TME in *Cdo1*^*fl/fl*^*Prrx1-cre*^*+*^ leukaemic mice as compared to the controls (Extended Data Fig. 5l–n), suggesting that leukaemia-driven remodelling of the bone marrow niche does not depend on taurine produced by osteolineage cells. We finally tested whether exogenous taurine supplements can promote leukaemia growth. Our experiments showed that the colony-forming ability of mouse LSCs and patient-derived AML cells increased by 1.2- to 3.3-fold in the presence of taurine (Extended Data Fig. 5o–q). Taurine supplements could significantly accelerate disease progression in immunocompetent mice (around threefold higher likelihood of death relative to the controls; Extended Data Fig. 5r), indicating that taurine can promote leukaemic progression.

Collectively, these data indicate a key role of taurine and APOE from the bone marrow niche in sustaining LSCs. As *SLC6A6* expression was significantly enriched in both human bcCML and AML LSCs as compared to the controls, we focused our studies on SLC6A6, as it may be broadly required for growth of aggressive leukaemias.

## TAUT loss impairs leukaemogenesis

**Fig. 3:**
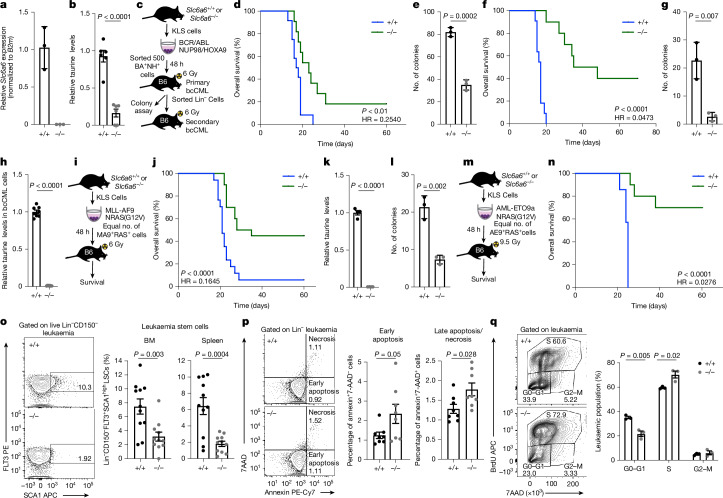
TAUT loss impairs myeloid leukaemia initiation and propagation in mouse models. **a**, Relative *Slc6a6* mRNA expression in whole bone marrow cells from *Slc6a6*^+/+^ (+/+) and *Slc6a6*^−/−^ (−/−) mice. Data are mean ± s.d. *n* = 3 replicates per cohort. **b**, Taurine in normal bone marrow cells. Data are mean ± s.e.m. *n* = 6 pelvic bones from three animals per cohort; data were combined from two independent experiments. **c**,**d**, The experimental strategy (**c**) and primary bcCML survival curve (**d**). *n* = 12 (+/+) and *n* = 11 (−/−). Data were combined from three independent experiments. **e**, CFU analysis of Lin^−^ bcCML cells from primary transplants. Data are mean ± s.d. *n* = 3 culture wells per cohort. **f**, Survival curve of secondary bcCML transplants. *n* = 11 (+/+) and *n* = 10 (−/−); data were combined from two independent experiments. **g**, CFU of Lin^−^ cells from secondary transplants. Data are mean ± s.d. *n* = 3 independent culture wells per cohort. **h**, Taurine in Lin^−^ LSCs. Data are mean ± s.e.m. *n* = 8 independent replicates per cohort from two independent samples. **i,j**, The experimental strategy (**i**) and survival curve (**j**) show the impact of TAUT loss on de novo MLL-AF9-driven AML. *n* = 17 (+/+) and n = 20 (−/−); data were combined from four independent experiments. **k**, Taurine in KIT^+^ AML cells. Data are mean ± s.e.m. *n* = 4 animals per cohort. **l**, CFU analysis of KIT^+^ AML cells. Data are mean ± s.d. *n* = 3 culture wells per cohort. **m,n**, Experimental strategy (**m**) and survival curve (**n**), showing the impact of TAUT loss on de novo AML-ETO9a-driven AML. *n* = 7 (+/+) and *n* = 10 (−/−). Data were combined from two independent experiments. **o**, Representative FACS plots and quantification of the Lin^−^CD150^−^SCA1^+^FLT3^+^ bcCML stem cell frequency in the bone marrow (left) and spleen (right) of recipients. Data are mean ± s.e.m. *n* = 11 animals per cohort. Data were combined from three independent experiments. **p**, Representative FACS plots and quantification of early apoptosis and necrosis in bcCML at 14 days after transplant. Data are mean ± s.e.m. *n* = 8 animals per cohort. Data were combined from two independent experiments. **q**, Representative FACS plots and graph showing the frequency of in vivo BrdU incorporation in bcCML. Data are mean ± s.e.m. *n* = 3 animals per cohort. Statistical analysis was performed using unpaired two-tailed Student’s *t*-tests (**b**, **e**, **g**, **h**, **k,** **l** and **o**–**q**) and log-rank tests (**d**, **f**, **j** and **n**). The mouse images in **c**, **i** and **m** are adapted from ref. ^6^, Springer Nature America. Source data

**Extended Data Fig. 6:**
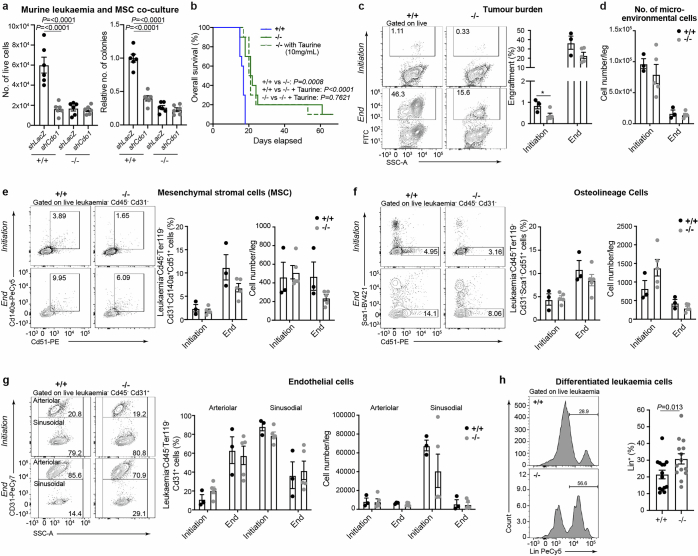
Impact of TAUT loss on leukaemia development in murine models. **a**, Number of live LSCs (left) and their CFU (right) post co-culture with leukaemic MSCs transduced with *shCdo1* or *shLacZ* (mean ± s.e.m.; n = 6 independent culture wells; data combined from 2 independent experiments; one-way ANOVA). **b**, Survival curve shows the impact of taurine supplements on murine leukaemia progression in irradiated recipients (n = 10 per cohort; data combined from two independent experiments; log-rank test). **c**, Representative FACS plots and quantification of BM engraftment in cancer at initiation and end point (mean ± s.e.m.; n = 3 +/+ and n = 5 −/−; data combined from two independent experiments). **d**-**g**, Graph (**d**) shows number of microenvironmental cells per mouse leg. Representative FACS plots and quantification of MSC (**e**), osteolineage (**f**), and endothelial (**g**) frequency (left) and total cell number (right) at initiation and end point (mean ± s.e.m.; n = 3 +/+ and n = 5 −/−; data combined from two independent experiments). **h**, Representative FACS histogram and quantification of Lin^+^ frequency in bcCML BM (mean ± s.e.m.; n = 14 animals per cohort; data combined from three independent experiments; **c, e**-**h** unpaired two-tailed Student’s t-test). Source data

To determine whether TAUT has a functional role in leukaemia progression we used global *Slc6a6*-knockout mice^25^ (Fig. 3a,b). While *Slc6a6*-knockout mice are born at Mendelian ratios, they can develop ageing-related defects in bone mass and retinal degeneration^25–27^. Our experiments showed that TAUT loss in LSCs significantly reduced their ability to grow when co-cultured with osteolineage cells expressing either control shRNA or shRNA against *Cdo1* (shCdo1; Extended Data Fig. 6a), indicating that they could not respond to taurine being produced by the niche. TAUT loss impaired initiation of bcCML in mouse models (3.9-fold higher likelihood of survival; Fig. 3c,d), which could not be rescued with taurine supplements (Extended Data Fig. 6b). TAUT loss also led to functional depletion in bcCML LSCs, as indicated by a 2.3-fold reduction in their colony-forming ability (Fig. 3e), as well as a marked increase in the survival of mice transplanted with *Slc6a6*^−/−^ LSCs (40%) relative to control LSCs (0%) in secondary transplant assays (21.2-fold higher likelihood of survival; Fig. 3f). The 8.5-fold reduction in colony-forming ability of bcCML LSCs from secondary transplants suggests that these leukaemias remain dependent on TAUT expression and taurine uptake for their continued propagation (Fig. 3g,h). We noted no significant differences in bone marrow microenvironmental populations of mice bearing *Slc6a6*^−/−^ leukaemias as compared to the controls, indicating that leukaemia-driven niche remodelling possibly reflects the extent of the tumour burden, and may be independent of taurine levels within leukaemia cells (Extended Data Fig. 6c–g).

To determine whether TAUT is broadly required for de novo AML growth, we tested the impact of its loss on initiation of leukaemia driven by MLL-AF9/NRAS(G12V) and by AML-ETO9a/NRAS(G12V). Our experiments showed that TAUT loss markedly delays the initiation of MLL-driven AML relative to the control (6.1-fold higher likelihood of survival; Fig. 3i–k). *Slc6a6*^*−/−*^ KIT^+^ AML LSCs from established disease formed 2.9-fold fewer colonies compared with the controls (Fig. 3l), indicating that TAUT loss depleted functional LSCs. Consistent with a key role of TAUT expression in myeloid leukaemia initiation, TAUT loss in disease driven by AML-ETO9a resulted in a marked increase in survival (70%) relative to the controls (0%) (36.3-fold higher likelihood of survival; Fig. 3m,n). At the cellular level, TAUT loss led to a 2.4- to 3.6-fold reduction in primitive Lin^−^CD150^−^FLT3^+^SCA1^+^ LSCs^6^ (Fig. 3o). Furthermore, TAUT loss increased necrosis and the Lin^+^ differentiated cells (Fig. 3p and Extended Data Fig. 6h) and promoted cell proliferation (Fig. 3q). These data demonstrate a critical requirement for TAUT in the initiation, self-renewal and propagation of myeloid leukaemia.

**Extended Data Fig. 7:**
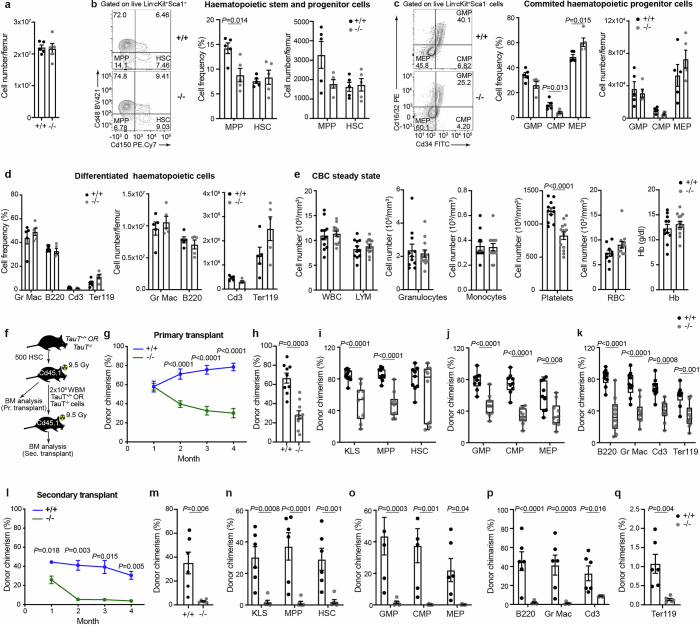
The role of TAUT in normal haematopoietic stem cell function. **a**, Average number of BM cells in +/+ and −/− mice (mean ± s.e.m.; n = 5 animals per cohort). **b**-**d** Representative FACS plots, and quantification of frequency (left) and cell number (right) of hematopoietic stem cells (**b**, HSC: Lin^−^cKit^+^Sca^+^CD150^+^CD48^−^) and multipotent progenitors (**b**, MPPs: Lin^−^cKit^+^Sca^+^CD150^−^CD48^−^), committed granulocyte–macrophage progenitors (**c**, GMP: Lin^−^IL7Ra^−^Kit^+^Sca1^−^CD34^+^CD16/3^+^), common myeloid progenitors (**c**, CMP: Lin^−^IL7Ra^−^Kit^+^Sca1^−^CD34^+^CD16/32^−^), megakaryocyte–erythroid progenitors (**c**, MEP: Lin^−^IL7Ra^−^Kit^+^Sca1^−^CD34^−^CD16/32^−^), and differentiated haematopoietic cells (**d**) in the BM of +/+ and −/− mice (mean ± s.e.m.; n = 5 animals per cohort; data combined from four independent experiments). **e**, Complete blood count of indicated cells and haemoglobin content in the peripheral blood of age and sex-matched 8-week-old littermates (mean ± s.e.m.; n = 11 +/+ and n = 13 −/−; data combined from two independent experiments). **f-g**, Experimental strategy (**f**), donor engraftment in peripheral blood over time (**g**). **h-k**, Average donor chimerism (**h**), frequency of KLS (Lin^−^cKit^+^Sca^+^), HSCs, and MPPs (**i**), committed progenitors GMP, CMP, and MEP (**j**) and, differentiated haematopoietic cells (**k**) in primary recipient BM four months post-transplant (**f-k**: mean ± s.e.m.; n = 9 +/+ and n = 10 −/−; data combined from two independent experiments). **l**, Donor engraftment in peripheral blood of secondary recipients (mean ± s.e.m.; n = 6 animals per cohort; **g, l**, two-way ANOVA). **m-q**, Average donor chimerism (**m**), frequency of KLS, HSCs, and MPPs (**n**), committed progenitors GMP, CMP, and MEP (**o**), and differentiated haematopoietic cells (**p, q**) in BM of secondary recipients four months post HSC transplant (mean ± s.e.m.; n = 6 animals per cohort; **a**-**e**, **h**-**k, m**-**q** unpaired two-tailed Student’s t-test). The mouse images in **f** are adapted from ref. ^6^, Springer Nature America. Source data

We next tested the impact of TAUT loss on normal haematopoiesis. Our analysis indicates that TAUT loss does not severely impact bone marrow cellularity, or the total numbers of HSCs, progenitors and differentiated cells at steady-state (Extended Data Fig. 7a–e). While TAUT loss did not impair initial HSC engraftment (1 month after transplant), donor chimerism dropped over time (Extended Data Fig. 7f,g). Although overall bone marrow chimerism 4 months after transplant was 2.4-fold lower in *Slc6a6*^*−/−*^ HSC recipients as compared to *Slc6a6*^*+/+*^ HSC recipients, these *Slc6a6*^*−/−*^ HSCs were able to contribute to all haematopoietic lineages (Extended Data Fig. 7h–k). Serial transplantation of *Slc6a6*^*+/+*^ and *Slc6a6*^*−/−*^ bone marrow cells showed a similar loss in *Slc6a6*^*−/−*^ engraftment over time (Extended Data Fig. 7l–q). These results suggest that, while TAUT loss does not impair steady-state haematopoiesis, it can impact long-term HSC self-renewal and maintenance. These data are consistent with previous studies showing that genetic loss of LSC-enriched genes such as *CD98*^1^, *STAU2*^6^, *MSI2*^28,29^, *BRD4*^30^ and *BCL2*^31^ can impair HSC self-renewal. However, therapeutic inhibition of CD98 showed minimal toxicity in AML phase I trials^1^ and BCL2 inhibitors are approved for AML therapy. It is therefore possible that non-genetic approaches using small-molecule inhibitors or gene silencing may identify a therapeutic window for TAUT targeting in human cells. We therefore tested the impact of TAUT inhibition on growth and proliferation of normal human HSPCs as well as patient-derived AML cells.

## TAUT is essential for human AML growth

**Fig. 4:**
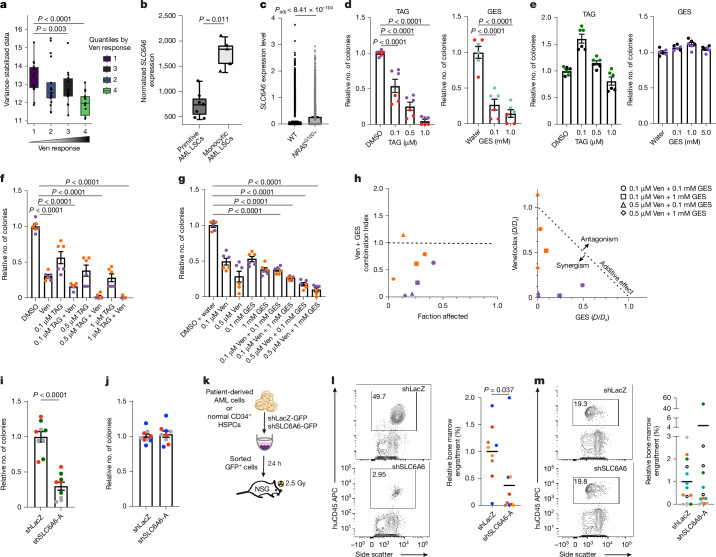
TAUT inhibition impairs growth of patient-derived AML cells. **a**, *SLC6A6* expression based on venetoclax (Ven) response (BEAT-AML). *n* = 193; dot plots show individual patients. Statistical analysis was performed using the DESeq2 log-rank test. **b,** *SLC6A6* expression in primitive and monocytic ROS^low^ AML LSCs^32^ (Gene Expression Omnibus (GEO): GSE132511). *n* = 7 (primitive) and *n* = 5 (monocytic). For the box plots in **a** and **b**, the centre line shows the median, the box limits show the interquartile range and the whiskers represent 1.5 × interquartile range. **c**, *SLC6A6* expression of human *NRAS*^G12D+^ versus wild-type cells (GEO: GSE253715). Statistical analysis was performed using the Seurat Findmarker function with the Wilcoxon rank-sum test^33^. **d**,**e**, CFU analysis of primary human AML (**d**) or normal human CD34^+^ bone marrow HSPCs (**e**) treated with dimethyl sulfoxide (DMSO)/water (control) or the indicated doses of TAG and GES. For **d** and **e**, data are mean ± s.e.m. *n* = 3 independent culture wells per sample from two independent samples; each colour represents a sample. **f**,**g**, CFU analysis of human AML cells treated with DMSO/water (control) or venetoclax (ABT-199) in combination with the indicated doses of TAG (**f**) or GES (**g**). Data are mean ± s.e.m. *n* = 3 independent culture wells per sample from two independent primary human AML samples. Each colour represents a sample. **h**, The combination index of GES and venetoclax calculated per fraction affected (left) and the normalized isobologram (right), as determined using the Chou–Talalay method. *n* = 2 independent primary human AML samples. Colours represent independent samples; shapes represent indicated venetoclax and GES combinations. *D*, drug dose; *D*_*x*_, median-effect dose. **i**,**j**, CFU analysis of primary human AML cells (**i**) or normal CD34^+^ HSPCs (**j**) that were transduced with lentiviral shRNAs targeting *LacZ* (control) or human *SLC6A6*. Data are mean ± s.e.m. *n* = 3 independent culture wells from *n* = 3 independent primary human patient samples. Each colour represents an independent sample. **k**–**m**, Experimental strategy (**k**) and representative FACS plots and graph, showing bone marrow engraftment of primary human AML (**l**) or primary human normal CD34^+^ HSPC (**m**) cells. The black lines show the mean. *n* = 9 animals per cohort from *n* = 4 independent primary human AML samples (**l**); and *n* = 13 animals per cohort from n = 5 independent normal human CD34^+^ HSPC samples (**m**). Each dot represents an animal; each colour represents an independent sample. Statistical analysis was performed using two-sample Wilcoxon tests (**b**), one-way ANOVA (**d**–**g**) and unpaired two-tailed Student’s *t*-tests (**i**, **j**, **l** and **m**). The mouse and culture well images in **k** are adapted from ref. ^6^, Springer Nature America. Source data

**Extended Data Fig. 8:**
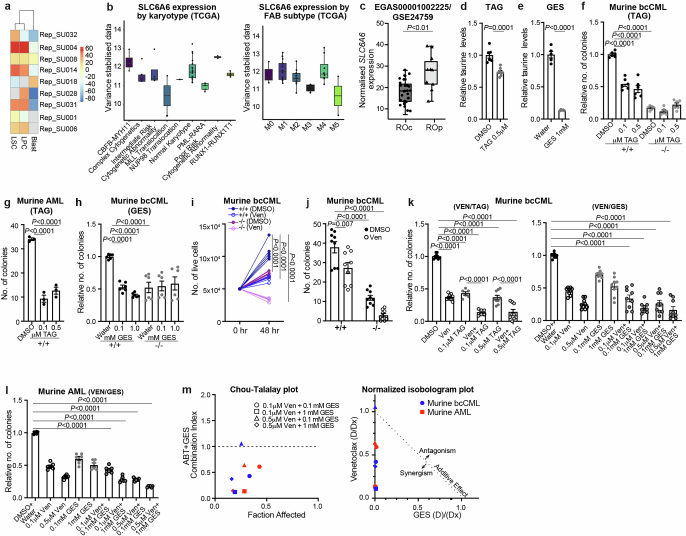
Taurine inhibition synergizes with venetoclax in vitro. **a**, SLC6A6 expression in human AML stem cells, progenitors and mature blasts (Gene Expression Commons). **b**, SLC6A6 expression by karyotype or FAB subtype (TCGA-AML, n = 146 karyotype and n = 149 FAB subtype; dot represents an individual patient). **c**, *SLC6A6* expression in relapse origin-committed (RO_C_) and relapse origin-primitive (RO_P_) AML^34^ (n = 29 RO_C_ and n = 10 RO_P_); (**b**-**c** central line, box, and whiskers represent median, interquartile range, minimum/maximum point within 1.5*IQR respectively; unpaired two-samples Wilcoxon test). **d**, Taurine in Lin^−^ bcCML cells treated with DMSO or TAG (mean±s.e.m.; n = 8 independent wells combined from 2 experiments). **e**, Taurine in Lin^−^ bcCML cells treated with GES or water (mean±s.e.m.; n = 5 independent culture wells; **d**-**e** unpaired two tailed Student’s t-test). **f**-**h**, CFU of Lin^−^ bcCML (**f**), ckit^+^ AML LSC (**g**) with DMSO or indicated doses of TAG, or Lin^−^ bcCML with water or indicated doses of GES (**h**)(mean±s.e.m.; n = 3 independent culture wells per cohort; data combined from two independent experiments for **f,h**). **i, j**, Viability (**i**) and CFU (**j**) of murine Lin^−^ bcCML cells treated with 0.5 mM venetoclax (ABT-199, Ven) for 48 h (n = 3 independent culture wells per cohort; data combined from two (**i**) or three (**j**) independent experiments; two-way ANOVA). **k**, CFU of Lin^−^ bcCML cells with DMSO/water, Ven, or indicated doses of TAG (left) and GES (right), or indicated combinations (mean ± s.e.m.; n = 3 per independent culture wells; data combined from 2 or 3 independent experiments). **l**, CFU of cKit^+^ AML LSC with DMSO/water, Ven, GES, or indicated combinations (mean±s.e.m.; n = 3 independent culture wells; data combined from 2 independent experiments, **f**-**h**, **j**-**l** one-way ANOVA). **m**, Combination index of GES and Ven per fraction affected (left) and isobologram (right) by Chou-Talalay method (n = 3 independent culture wells; data combined from 3 independent experiments; colours represent sample type, shapes represent Ven/GES combinations). Source data

Our analyses of available gene expression datasets indicate that *SLC6A6* expression is enriched in leukaemia stem/progenitors as compared to more mature blasts (Extended Data Fig. 8a). While *SLC6A6* is broadly expressed in AML irrespective of karyotype or FAB subtype (TCGA; Extended Data Fig. 8b), increased expression correlates with venetoclax resistance (BEAT-AML; Fig. 4a). *SLC6A6* expression is also enriched in reactive oxygen species (ROS)-low LSCs from human monocytic AMLs (subtype clinically associated with venetoclax resistance; CD45^bright^SSC^high^CD117^−^CD11b^+^CD68^+^) compared with primitive AML (Fig. 4b; CD45^med^SSC^low^CD117^+^CD11b^−^CD68^−^)^32^. We find increased *SLC6A6* expression in leukaemia cells carrying RAS mutations as compared to wild-type cells, consistent with data correlating RAS mutations with venetoclax resistance^33^ (Fig. 4c). *SLC6A6* is also highly expressed in relapsed AML originating from stem/progenitor like cells compared with more committed populations^34^ (Extended Data Fig. 8c). These data collectively indicate that inhibiting *SLC6A6* may be of value across AML subtypes, including those commonly associated with venetoclax resistance.

To determine whether small-molecule inhibitors of TAUT can effectively block leukaemia growth, we used two well-characterized structural analogues of taurine that inhibit uptake: 6-aminomethyl-3-methyl-4H-1,2,4-benzothiadiazine-1,1-dioxide hydrochloride^35^ (TAG) and guanidinoethyl sulphonate^36^ (GES; Extended Data Fig. 8d,e). TAG and GES treatment led to a substantial reduction in colonies formed by *Slc6a6*^*+/+*^ mouse LSCs but not *Slc6a6*^*−/−*^ cells (Extended Data Fig. 8f–h). Importantly, while TAG and GES impaired growth of primary human AML cells in colony assays by 1.8- to 20-fold, they did not impact normal human CD34^+^ HSPC colony growth (Fig. 4d,e). We next tested whether venetoclax can exacerbate the impact of TAUT loss and/or inhibition on LSC function. Our experiments showed that venetoclax reduced the viability of mouse *Slc6a6*^*−/−*^ LSCs by 3.2-fold and lowered their colony formation by 13.2-fold as compared to the controls (Extended Data Fig. 8i,j). While TAG, GES or venetoclax alone could impair mouse LSC colony formation by 1.4- to 3.9-fold, their combination was synergistic and led to 2.3- to 7.2-fold fewer colonies as compared to the controls (Extended Data Fig. 8k–m). While primary human AML cells that were treated with GES, TAG or venetoclax alone formed 1.3- to 8.3-fold fewer colonies as compared to the controls, combining the treatments substantially impaired colony formation by 2.4- to 150-fold. These data indicate that taurine inhibitors can synergize with venetoclax in blocking the growth of human AML cells (Fig. 4f–h).

**Extended Data Fig. 9:**
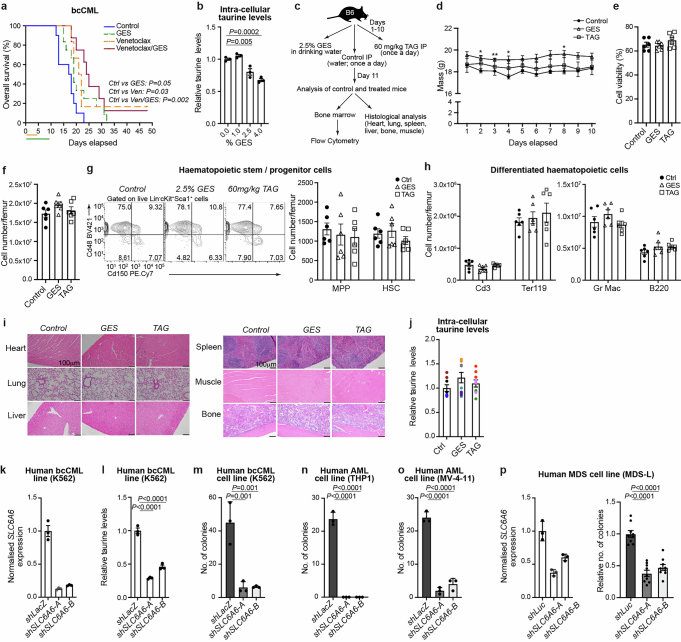
Taurine inhibition in vivo. **a**, Survival curve showing the impact of treating mice transplanted with Lin^−^ bcCML cells with GES (2.5%), Ven (50 mg/kg), or their combination (n = 10 control, n = 12 GES, n = 6 Ven, n = 8 GES/Ven); data combined from three independent experiments; lines below graph represent days of treatment; log-rank test). **b**, Taurine in 10^7^ BM cells from leukaemic mice treated with indicated amounts of GES for 13–16 d (mean ± s.e.m.; n = 3 technical replicates per cohort). **c, d**, Experimental strategy (**c**) and mass (**d**) of mice during treatment with GES, TAG, or control (mean ± s.e.m.; n = 3 animals per cohort; two-way ANOVA). **e,f**, Viability (**e**) and average number of BM cells (**f**) (mean ± s.e.m.; n = 6 mice per cohort; data combined from two independent experiments). **g-h**, Representative FACS plots (left), and number (right) of HSC and MPP (**g**), and differentiated haematopoietic cells (**h**) (mean ± s.e.m.; n = 6 animals per cohort; data combined from two independent experiments; two-way ANOVA). **i**, Haematoxylin & Eosin staining of indicated tissues in treated animals. **j**, Intracellular taurine levels by LC/MS in BM cells from treated animals (mean ± s.e.m.; n = 8 independent replicates from n = 3 control, n = 10 from 4 GES, n = 9 from 3 TAG treated animals; each colour represents a mouse). **k, l**, *SLC6A6* expression (**k**) and taurine levels (**l**) in K562 cells transduced with shRNAs targeting *LacZ* and *SLC6A6* (mean ± s.d.; n = 3 technical replicates per cohort). **m**-**o**, CFU of human leukaemia cell lines transduced with lentiviral shRNAs targeting *LacZ* or *SLC6A6*, K562 (**m**), THP1 (**n**), and MV-4-11 (**o**) (mean ± s.d.; n = 3 independent culture wells per cohort). **p**, Relative *SLC6A6* expression (left; mean ± s.d.; n = 3 technical replicates per cohort) and CFU (right) in MDS-L cells transduced with shRNAs targeting *Luc* and *SLC6A6* (mean ± s.e.m.; n = 9 independent culture wells per cohort; data combined from 3 independent experiments; **b**, **e**-**h, j**, **l**-**p** one-way ANOVA). The mouse image in **c** is adapted from ref. ^6^, Springer Nature America. Source data

As TAG and GES did not effectively block taurine uptake in vivo (Extended Data Fig. 9a–j), we used shRNA-based approaches to determine the impact of inhibiting TAUT expression on human AML growth. Knocking down *SLC6A6* expression using two independent shRNAs significantly impaired taurine uptake by 2.2 to 3.4-fold, as well as the colony-forming ability of human bcCML, AML and MDS cell lines by 2- to 12-fold (Extended Data Fig. 9k–p). Our experiments showed that *SLC6A6* knockdown in samples from patients with AML reduced their colony-forming ability by 2.33- to 9.12-fold as compared to the controls (Fig. 4i). By contrast, *SLC6A6* knockdown did not impact colonies formed by normal human CD34^+^ HSPCs (Fig. 4j). Importantly, while *SLC6A6* knockdown reduced the engraftment of primary AML cells by 1.2- to 40-fold in patient-derived xenograft models (Fig. 4k,l), it did not significantly impair engraftment of normal human CD34^+^ HSPCs in xenograft models (Fig. 4m).

Collectively, our data identify a critical requirement for *SLC6A6* expression in primary human AML growth and indicate that blocking taurine transport may be of value in aggressive myeloid leukaemias.

## Taurine drives glycolysis in leukaemia

**Fig. 5:**
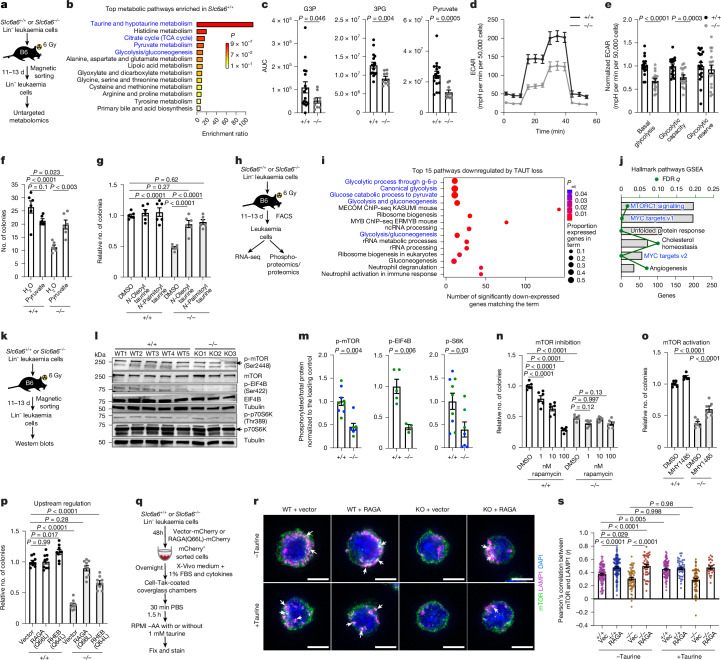
TAUT loss impairs glycolysis and mTOR signalling in myeloid leukaemia cells. **a**, The untargeted metabolomics strategy. **b**, Unbiased Enrichr analysis (MetaboAnalyst; *P*_adj_ ≤ 0.05). TCA, tricarboxylic acid. **c**, Quantification of glycolysis-associated metabolites. Data are mean ± s.e.m. *n* = 16 samples from *n* = 6 *Slc6a6*^*+/+*^ leukaemic mice and *n* = 9 samples from *n* = 3 *Slc6a6*^*−/−*^ leukaemic mice. Statistical analysis was performed using unpaired Student’s *t*-tests with Welch’s correction. AUC, area under the curve; G3P, glyceraldehyde-3-phosphate; 3PG, 3-phosphoglyceric acid. **d**,**e**, Extracellular acidification (ECAR) curve (**d**) and quantification (**e**). Data are mean ± s.e.m. *n* = 18 independent culture wells per cohort from four bcCML samples. Data are combined from four independent experiments. **f**,**g**, CFU analysis of Lin^−^ bcCML cells supplemented with 1 mM pyruvate (**f**) or 80 μM cell-permeable taurine conjugates (**g**). Data are mean ± s.e.m. *n* = 3 independent culture wells per cohort. Data were combined from two independent experiments. **h**, Experimental strategy. **i**, Unbiased Enrichr analysis of transcriptomic data. ChIP–seq, chromatin immunoprecipitation followed by sequencing; g-6-p, glucose-6-phosphate; ncRNA, non-coding RNA. **j**, Gene sets significantly enriched in *Slc6a6*^*+/+*^ leukaemic mice. GSEA, gene set enrichment analysis. **k**–**m**, Schematic (**k**), immunoblot (**l**) and quantification (**m**) of the indicated proteins (Extended Data Fig. 11e). Data are mean ± s.e.m. *n* = 9 (*Slc6a6*^*+/+*^) and *n* = 7 (*Slc6a6*^*−/−*^) for p-mTOR and p-pS6K. Data were combined from two independent experiments, indicated by the two colours. *n* = 5 (*Slc6a6*^*+/+*^) and *n* = 3 (*Slc6a6*^*−/−*^) for p-EIF4B. For **d**, **e** and **m**, statistical analysis was performed using unpaired two-tailed Student’s *t*-tests. **n**,**o**, CFU analysis of Lin^−^ bcCML cells treated with rapamycin (**n**) or 2 μM mTOR activator MHY1485 (**o**). Data are mean ± s.e.m. *n* = 6 independent culture wells per cohort. Data were combined from two independent experiments. **p**, CFU analysis of Lin^−^ bcCML cells infected with vector, RAGA(Q66L) or RHEB(Q64L). Data are mean ± s.e.m. *n* = 9 independent culture wells per cohort. Data were combined from three independent experiments. **q**–**s**, The strategy to determine mTOR and LAMP1 co-localization in Lin^−^ bcCML cells infected with RAGA(Q66L) or vector (vec) with or without taurine (**q**), microscopy images (**r**) and analysis (**s**). *n* = 83 (*Slc6a6*^*+/+*^, vector, −taurine), 58 (*Slc6a6*^*−/−*^, vector, −taurine), 125 (*Slc6a6*^*+/+*^, RAGA, −taurine), 43 (*Slc6a6*^*−/−*^, RAGA, −taurine), 78 (*Slc6a6*^*+/+*^, vector, +taurine), 68 (*Slc6a6*^*−/−*^, vector, +taurine), 40 (*Slc6a6*^*+/+*^, RAGA, +taurine) and 23 (*Slc6a6*^*−/−*^, RAGA, +taurine). Data were combined from two independent experiments. mTOR and LAMP1 co-colocalization is indicated by white arrows. AA, amino acids; KO, knockout. For **f**, **g**, **n**–**p** and **s**, statistical analysis was performed using one-way ANOVA. Scale bars, 5 μm (**r**). Blue text in **b**, **i** and **j** represents pathways of interest. The mouse and culture well images in **a**, **h**, **k** and **q** are adapted from ref. ^6^, Springer Nature America. Source data

**Extended Data Fig. 10:**
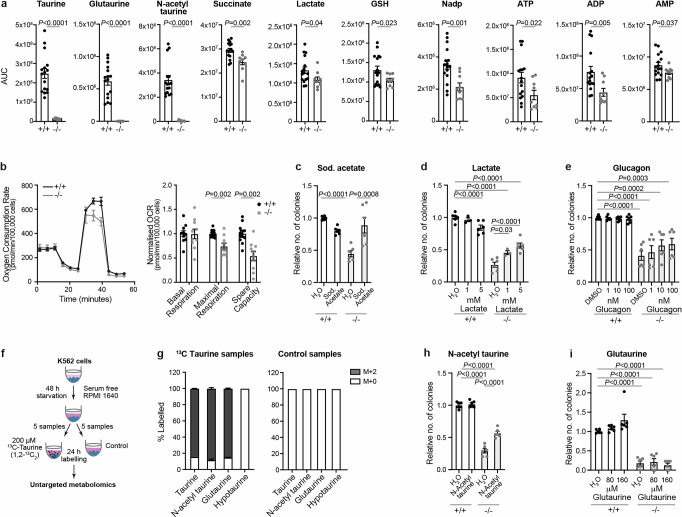
TAUT loss impairs energy metabolism in myeloid leukaemia. **a**, Quantification of taurine and glycolysis associated metabolites in +/+ and −/− Lin^−^ bcCML cells (mean ± s.e.m.; n = 16 samples from n = 6 +/+ leukaemic mice and n = 9 samples from n = 3 −/− leukaemic mice; unpaired two-tailed Student’s t-test with Welch’s correction). **b**, Curve and quantification of normalized of oxygen consumption rate (OCR) in Lin^−^ bcCML cells (mean ± s.e.m.; n = 10 independent culture wells per cohort from 3 bcCML samples; data combined from three independent experiments; unpaired two-tailed Student’s t-test). **c**-**e**, Impact of supplementing 5 mM sod. acetate (**c**), lactate (**d**), or indicated amounts of glucagon (**e**) on the CFU of Lin^−^ bcCML cells (mean ± s.e.m.; n = 3 independent culture wells per cohort; data combined from 2 independent experiments; one-way ANOVA). **f**, Experimental strategy used to determine ^13^C taurine tracing in K562 cells by untargeted metabolomics. **g**, Incorporation of ^13^C label from taurine in indicated metabolites (mean ± s.e.m.; n = 5 independent biological replicates). **h,i**, Colony-forming ability of Lin^−^ bcCML cells in the presence of 160 mM N-acetyl taurine (**h**) or indicated amounts of glutaurine (**i**) (mean ± s.e.m.; n = 3 independent culture wells per cohort; data combined from two independent experiments; one-way ANOVA). Source data

To establish the mechanisms by which taurine uptake in leukaemia cells promotes disease progression, we determined metabolic changes in the absence of taurine. Our untargeted metabolomic analysis of mouse LSCs identified significant downregulation of glycolysis/TCA related pathways and metabolites such as pyruvate, glyceraldehyde-3-phosphate and 3-phosphoglycerate in *Slc6a6*^*−/−*^ cells, suggesting that taurine may regulate energy metabolism (Fig. 5a–c and Extended Data Fig. 10a). Consistent with this, we noted a 1.3- to 1.8-fold reduction in basal glycolysis, glycolytic capacity, maximal oxygen consumption and spare respiratory capacity in *Slc6a6*^*−/−*^ LSCs as compared to the controls (Fig. 5d,e and Extended Data Fig. 10b). To functionally test the role of glycolysis and TCA associated metabolites, we determined whether bypassing these could rescue *Slc6a6*^*−/−*^ defects. Our experiments showed that the colony-forming ability of *Slc6a6*^*−/−*^ LSCs could be significantly rescued by pyruvate, sodium acetate and lactate, but not glucagon (Fig. 5f and Extended Data Fig. 10c–e). These data suggest that glycolysis, and not gluconeogenesis, is the primary downstream effector of taurine in leukaemia cells.

We next tested whether taurine contributed to any cellular metabolite by determining ^13^C-taurine incorporation in K562 leukaemia cells. Our untargeted metabolomic approach identified ^13^C label only in known taurine conjugates, *N*-acetyl taurine and glutaurine (Extended Data Fig. 10f,g). Membrane-permeable taurine fully rescued colonies formed by *Slc6a6*^*−/−*^ cells (Fig. 5g), and *N*-acetyl-taurine partly rescued this defect (Extended Data Fig. 10h), perhaps by breakdown to taurine and acetate^37^. By contrast, glutaurine was unable to rescue the *Slc6a6*^*−/−*^ colony formation (Extended Data Fig. 10i). These experiments indicate that taurine, and not a secondary metabolite, promotes leukaemia growth due to an indirect effect on glycolysis, possibly through downstream signalling.

**Extended Data Fig. 11:**
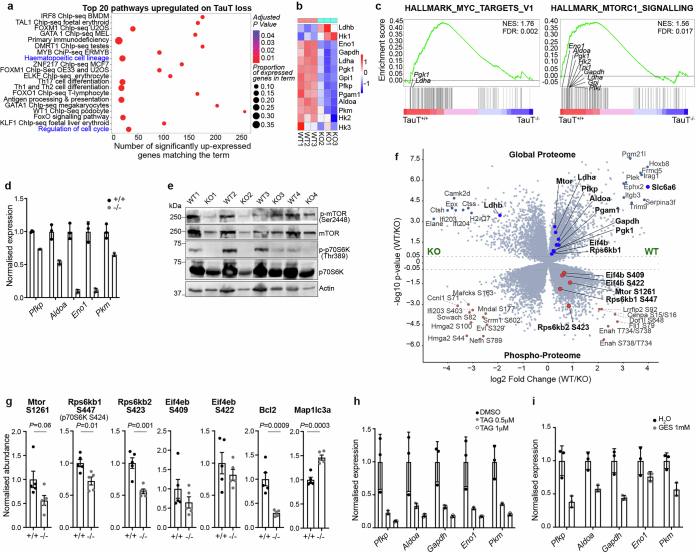
Transcriptomic and proteomic analysis of leukaemia in the absence of TAUT. **a**, Top 20 upregulated pathways in −/− time matched bcCML cells as compared to +/+ controls. **b**, Heatmap of glycolysis associated genes in bcCML samples. **c**, GSEA showing downregulation of genes associated with Myc and mTORC1 pathways on TauT loss (**a**-**c**, n = 3 animals per cohort). **d**, Relative expression of glycolysis associated genes in +/+ and −/− Lin^−^ bcCML cells (mean ± s.d.; n = 3 technical replicates per cohort). **e**, Western blot shows phospho-mTOR (pmTOR), mTOR, phosopho-p70S6k (p-p70S6K), p70S6K, and actin protein expression in +/+ and −/− Lin^−^ bcCML cells (also see Fig. 5k–m, n = 4 independent disease end point samples/cohort). **f**, Volcano plot of global proteome (upper panel) and phosphoproteome (bottom panel) with differential abundance in +/+ and −/− leukaemia cells from time matched recipients (n = 5 animals per cohort). **g**, Normalized abundance of mTOR pathway proteins (mean ± s.e.m.; n = 5 animals per cohort; unpaired two-tailed Student’s t-test). **h**, Expression of glycolysis related genes in +/+ Lin^−^ bcCML treated with indicated doses of TAG for 72 h (mean ± s.d.; n = 3 technical replicates per cohort). **i**, Expression of glycolysis related genes in +/+ Lin^−^ bcCML treated with 1 mM GES for 72 h (mean ± s.d.; n = 3 technical replicates per cohort). Source data

To identify signals downstream of taurine, we performed RNA-seq as well as phosphoproteome and total-proteome analyses of primary mouse leukaemia cells (Fig. 5h). Our RNA-seq analysis identified 932 downregulated and 1,158 upregulated genes in the absence of TAUT (*P*_adj_ < 0.05). The 1,158 upregulated genes included pathways associated with haematopoietic cell lineage and cell cycle regulation (Extended Data Fig. 11a,b). The 932 downregulated genes primarily constituted pathways associated with glycolysis (Fig. 5i and Extended Data Fig. 11b–d), consistent with reduced abundance of glycolysis associated metabolites in these cells (Fig. 5c). To determine effectors of glycolytic downregulation on TAUT loss, we performed gene set enrichment analysis. This identified profound downregulation of oncogenic MYC and mTOR pathways (Fig. 5j and Extended Data Fig. 11c).

**Extended Data Fig. 12:**
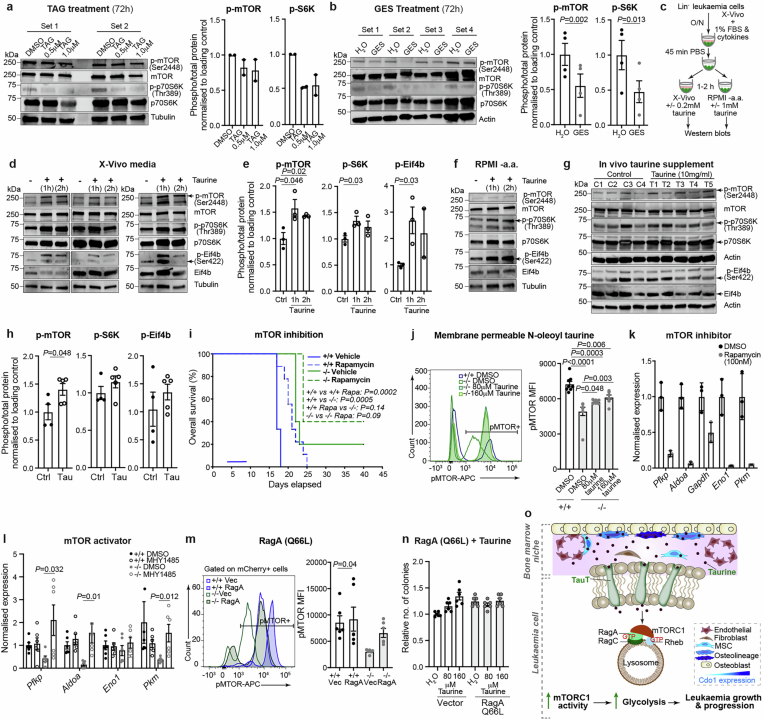
Taurine activates mTOR to promote leukaemia growth. **a,b**, Immunoblot and quantification of mTOR pathway proteins in +/+ Lin^−^ bcCML treated with indicated doses of TAG (**a**, mean ± s.d.; n = 2 replicates per cohort) or 1 mM GES (**b**, mean ± s.e.m.; n = 4 samples per cohort). **c**-**f**, Experimental strategy (**c**), immunoblot (**d, f**), and quantification (**e**) of indicated proteins in Lin^−^ bcCML cells +/− 200 mM taurine (mean ± s.e.m.; n = 3 biological replicates; gels processed in parallel; tubulin run on each gel as sample processing control). **g,h**, Immunoblots (**g**), and quantification (**h**) of mTOR pathway proteins in Lin^−^ bcCML cells from leukaemic mice supplemented with taurine for 10 days (mean ± s.e.m.; n = 4 control and n = 5 taurine; **b, e, h** unpaired two-tailed Student’s t-test). **i**, Survival curve shows impact of 5 mg/kg rapamycin treatment for 6 d on leukaemia progression. Line represents treatment days (n = 9 +/+ and n = 5 −/−; data combined from two independent experiments; log-rank test). **j**, Histogram and geometric Mean Fluorescence Intensity (MFI) of p-mTOR in Lin^−^ bcCML cells treated with DMSO or N-Oleoyl taurine for 36–40 h. (mean ± s.e.m.; n = 4 samples per cohort; data combined from two independent experiments). **k,l**, Expression of glycolysis genes in +/+ Lin^−^ bcCML cells treated with rapamycin for 24 h (**k**; mean ± s.d.; n = 3 technical replicates per cohort) or 2 mM MHY1485 for 48 h (**l**; mean ± s.e.m.; n = 3 technical replicates per cohort; data combined from two independent experiments). **m**, Histogram and MFI of p-mTOR in Lin^−^ bcCML cells infected with vector or RagA(Q66L) (mean ± s.e.m.; n = 6 samples per cohort; data combined from two independent experiments). **n**, CFU of Lin^−^ bcCML cells infected with vector or RagA_Q66L_ and treated with indicated amounts of taurine (mean ± s.e.m.; n = 3 independent culture wells per cohort; data combined from two independent experiments; **j**, **l**-**n** one-way ANOVA). **o**, Schematic shows taurine from BM osteolineage cells promotes RagA-dependent mTOR activation and glycolysis in leukaemia cells to drive disease progression. The culture well image in **c** is adapted from ref. ^6^, Springer Nature America. Source data

As mTOR signalling is known to regulate expression of glycolysis-related genes^38^, we tested whether TAUT loss impairs mTOR activation. Western blot analysis showed that TAUT loss results in a 2.2- to 3-fold reduction in activated mTOR, p70S6k and pEIF4B as compared to the controls (Fig. 5k–m and Extended Data Fig. 11e). Our global phosphoproteome and total-proteome analyses also showed reduced expression of phosphorylated proteins associated with mTOR signalling, as well as 3.3-fold reduced BCL2 and 1.5-fold increased LC3A expression in the absence of TAUT (Extended Data Fig. 11f,g). Like TAUT loss, TAUT inhibition with TAG and GES downregulated glycolysis-related gene expression (Extended Data Fig. 11h,i) and impaired phosphorylation of mTOR signalling (Extended Data Fig. 12a,b). Consistent with a direct impact of taurine on mTOR signalling, acute treatment in vitro and long-term taurine dosing in vivo could activate mTOR signalling (Extended Data Fig. 12c–h). Inhibiting mTOR with rapamycin impaired the growth of wild-type cells but did not impact *Slc6a6*^*−/−*^ cells in vitro or in vivo (Fig. 5n and Extended Data Fig. 12i,k). By contrast, mTOR activation with MHY1485^39^ rescued the colony-forming ability of *Slc6a6*^*−/−*^ LSCs to 60% of the control, and membrane-permeable taurine rescued mTOR activation (Fig. 5o and Extended Data Fig. 12j–l). Collectively, these data indicate that mTOR signalling has a key role downstream of taurine in leukaemia cells.

Amino acid availability can be sensed by RAG GTPases to facilitate mTORC1 localization to the outer lysosomal membrane^40^ and its interaction with RHEB GTPases^41^, thereby promoting mTORC1 activation. We hypothesized that taurine may activate mTOR by promoting its interaction with RAG GTPases. To test this, we determined the effect of expressing constitutively active RAGA(Q66L) or RHEB(Q64L) on the colony-forming ability of *Slc6a6* wild-type and null cells. Our experiments showed that, while RHEB(Q64L) partially rescued *Slc6a6*^*−/−*^ colony formation, this could be fully rescued by RAGA(Q66L) (Fig. 5p). Furthermore, mTOR phosphorylation in *Slc6a6*^*−/−*^ cells could be rescued by RAGA(Q66L) (Extended Data Fig. 12m). Finally, we directly tested whether taurine could promote mTOR interactions with lysosomes in wild-type and *Slc6a6*^*−/−*^ leukaemia cells expressing RAGA(Q66L) (Fig. 5q). Our immunofluorescence-based analysis showed that, at the baseline, *Slc6a6*^*−/−*^ cells have 1.2-fold lower mTOR–LAMP1 interaction, which could be fully rescued by RAGA(Q66L). Taurine supplements increased mTOR–LAMP1 co-localization to levels seen in cells expressing RAGA(Q66L). However, this interaction could not be increased further by adding taurine to RAGA(Q66L)-expressing cells (Fig. 5r,s), consistent with our colony-forming assays (Extended Data Fig. 12n).

Collectively, our studies show that the taurine–TAUT axis regulates glycolysis in myeloid leukaemia cells by promoting RAG-mediated activation of mTOR signalling.

## Discussion

We use scRNA-seq to define the changing landscape of the non-immune cancer microenvironment with disease progression, and to identify unique niche driven signals that promote disease progression. scRNA-seq has been effectively used to characterize the immune microenvironment of both solid tumours and leukaemias^20,42–45^. Despite technical limitations in detecting non-immune stromal cells, a few studies have identified antigen-presenting fibroblasts and immune-suppressive endothelial cells in lung and pancreatic cancers^46–48^. A reduction in osteoblasts has been noted during leukaemia initiation in bone marrow niche damaged by irradiation^13^. However, dynamic changes in the TME during leukaemia progression have not been defined.

Our temporal scRNA-seq based TME analysis identifies an expansion in MSCs and their immature osteo-associated progeny, along with a loss in mature osteo-associated cells. This skew in osteolineage populations possibly results from downregulation of MSC differentiation signals during leukaemia progression, and may explain conflicting findings using candidate osteoblast markers^49,50^. Our data indicate that temporal expansion in arteriolar endothelial cells is accompanied by a loss in signals that are essential for endothelial cell integrity and function, consistent with leaky blood vessels seen by in vivo imaging of the AML niche^1,3,51^. In addition to population-level changes, our studies identify signals such as KIT, thrombospondin, taurine and apolipoproteins from the TME that are essential for cancer progression. While multiple TME ligands such as APOE and PVR are detected through the disease trajectory, the populations expressing these can change over time. Thus, therapeutic approaches aimed at targeting TME-driven signals may be more effective than blocking TME remodelling or inhibiting individual stromal populations.

Consistent with clinicopathological similarities between AML and bcCML^52^, our gene expression analysis of human AML and bcCML CD34^+^ LSCs identifies distinct overlap. Our unbiased approach to determine LSC-enriched cell surface receptors that are essential for disease progression identified multiple genes that are known to be critical for AML, including CD96^53^, CD47^54^ and protein kinase D2^55^. Although these are required for leukaemic progression, they are not associated with poor prognosis (TCGA-LAML), in contrast to *LDLR* and *SLC6A6* that we describe here. It is therefore possible that other cell surface receptors identified by our analysis also have a functional role in disease progression and should be explored further to determine new therapeutically relevant signals. As the APOE–LDLR and taurine–TAUT axes that we identify are known to have a role in ageing^27,56^, it is possible that cancer-associated signals in our TME–LSC interactome may be of broad relevance in ageing-related disorders such as MDSs, as we see with TAUT.

Biosynthesis of taurine from cysteine is known to occur in the liver, kidneys, adipose tissues and pancreas^24^. Our data identify bone marrow osteolineage cells as a novel source of taurine in the leukaemia niche. Our studies blocking taurine produced by osteolineage cells establish a key role of TME-driven taurine synthesis in LSC survival and self-renewal. However, it is possible that taurine or β-alanine produced outside the bone marrow niche also contribute to disease progression. Consistent with this, our data suggest that taurine supplements can accelerate myeloid leukaemia progression in mouse models. As taurine is a common ingredient in energy drinks, and is often provided as a supplement to mitigate the side-effects of chemotherapy^17^, our work suggests that it may be of interest to carefully consider the benefits of supplemental taurine in patients with leukaemia.

Mechanistically, we identify a critical requirement of taurine from the microenvironment in regulating mTOR-driven glycolysis in leukaemia cells (Extended Data Fig. 12o). We can rescue the growth of *Slc6a6-*null LSCs by circumventing glycolysis with pyruvate or by ectopically activating mTORC1 using GTP-bound RAGA mutants. Our data showing that constitutively active Rag-GTPases can rescue mTOR interactions with lysosomes, and rescue mTOR phosphorylation, in *Slc6a6*^*−/−*^ LSCs indicate that taurine levels in leukaemia cells may be detected by hitherto unidentified sensors, like those for arginine and leucine^57,58^. Our in vivo data showing a strong impact of targeting taurine uptake using genetic approaches in AML suggest that it would be of considerable interest to develop stable and effective in vivo inhibitors of taurine in future studies. In light of early clinical success of glutamine inhibitors in MDS and AML^59,60^, our work suggests that evaluating taurine-transport inhibitors in normal and leukaemic cells may be of therapeutic interest.

## Methods

### Generation of experimental mice

The *Slc6a6* mice were bred as described previously^25^. For all mouse leukaemia experiments, male and female *Slc6a6*^*+/+*^ and *Slc6a6*^*−/−*^ mice were used as donors and B6-CD45.1 (*B6.SJL-Ptprc*^*a*^*Pepc*^*b*^*/BoyJ*) or C57BL6/J mice were used as transplant recipients. For xenograft experiments with human cells, NSG mice (*NOD.Cg-Prkdcscid Il2rgtm1Wjl/SzJ*) were used as transplant recipients. All mice were 6–16 weeks of age. All animals used for in vivo studies were randomly selected to receive either control or treatments. Animals were selected based on genotype, age and sex. No statistical method was used to predetermine sample size for experiments. Adequate sample size was determined based on previous publications^1,3,6^. Mice were bred and maintained in the animal care facilities at the University of Rochester. All animal experiments were performed according to protocols approved by the University of Rochester’s Committee on Animal Resources. Mice were housed with the same sex in ventilated cages, under a 12 h–12 h light–dark cycle with temperature (64–79 °F) and humidity (winter levels, <30%; summer levels, >70%) control. Mice were given enrichment material and fed a standard chow diet. Irradiated recipients were maintained on acid water HYDROPAC (pH 2.5–3.0) and irradiated sulfatrim diet (Mod LabDiet 5P00 with 0.025% trimeth and 0.124% sulfameth). NSG recipients were housed in BSL-2 facility. *Cdo1*^*fl/fl*^ mice were generated in collaboration with the Transgenic and Genome Editing Core Facility at Augusta University using CRISPR–Cas9 gene editing. These mice were bred to the MSC-specific *Prrx1-cre* (*B6.Cg-Tg(Prrx1-cre)1Cjt/J*) to generate a *Cdo1*^*fl/fl*^*Prrx1-cre*^*+*^ mice, where taurine production is blocked in the MSC/osteolineage cells. As *Prrx1*-Cre can be expressed in the female germline, only male *Prrx1-cre*^*+*^ mice were used for breeding and experiments. Blinding was not relevant to mouse experiments as researchers needed to know the conditions for each experiment. Flow cytometry, FACS, western blots, Seahorse analysis, sequencing and imaging used analytical machines and blinding was therefore not necessary.

### Cell isolation and FACS analysis

Cells were suspended in Hanks’ balanced salt solution (Gibco) with 5% FBS and 2 mM EDTA. Cells were prepared for FACS analysis and sorting as previously described^1,6^. Antibodies used for defining haematopoietic cell populations were as follows: CD3ε, CD4, CD8, GR1, CD11b, TER119, CD45R and CD19 (all for lineage), KIT, SCA1, CD48 and CD150. All antibodies were purchased from, eBioscience, BioLegend or BD Biosciences. A detailed list of antibodies is provided in Supplementary Table 2. Analysis was performed on LSRFortessa (BD Biosciences), and cell sorting was performed on the FACSAria II (BD Biosciences). Data were analysed using FlowJo.

### Retroviral and lentiviral constructs and virus production

Retroviral MSCV-BCR-ABL-IRES-GFP (or -tNGFR) and MSCV-NUP98-HOXA9-IRES-YFP (or -huCD2 and -tNGFR) were used to generate bcCML. AML was generated with MSCV-MLL-AF9-IRES-tNGFR and MSCV-NRAS(G12V)-IRES-huCD2 or MSCV-AML-ETO9a and MSCV-NRAS(G12V)-IRES-YFP. RAGA (Q66L; Addgene, 99712) and RHEB (Q64L; Addgene, 64607) were cloned into the MSCV-mCherry backbone (Addgene, 52114). Lentiviral shRNA constructs were designed and cloned into the pLV-hU6-EF1a-green or pLV-hU6-EF1a-red backbone (Biosettia) according to the manufacturer’s protocol. A detailed list of shRNA sequences is provided in Supplementary Table 1. Virus was produced in 293T cells (ATCC) transfected with viral constructs along with VSV-G, Gag-Pol (retroviral production) or pRSV-rev, phCMV and pMDlg/pRRE (lentivirus production) using X-tremeGENE-HP reagent (Roche). Viral supernatants were collected for 3 to 6 days followed by ultracentrifugal concentration at 20,000 rpm for 2 h.

### Generation and analysis of leukaemia models

Bone marrow KLS cells were sorted from *Slc6a6*^*+/+*^ *or* *Slc6a6*^−*/*−^ mice and cultured overnight in X-VIVO15 (Lonza) medium supplemented with 10% FBS (GeminiBio), 50 μM 2-mercatpoethanol, SCF (100 ng ml^−1^, R&D Systems), TPO (10 ng ml^−1^, R&D Systems) and penicillin–streptomycin (Gibco). Cells were retrovirally infected with MSCV-BCR-ABL-IRES-GFP (or -tNGFR) and MSCV-NUP98-HOXA9-IRES-YFP (or -huCD2 or -tNGFR) to generate bcCML. AML was generated by sorting bone marrow KLS cells from *Slc6a6*^*+/+*^ or *Slc6a6*^−*/*−^ mice and culturing in RPMI medium (Gibco) supplemented with 20% FBS, 50 μM 2-mercaptoethanol, 100 ng ml^−1^ SCF (R&D Systems), 10 ng ml^−1^ IL-3, and 10 ng ml^−1^ IL-6 (R&D Systems). Cells were retrovirally infected with MSCV-MLL-AF9-IRES-tNGFR and MSCV-NRAS(G12V)-IRES-YFP cells were collected 48 h after infection, sorted by FACS for BCR-ABL^+ and^ NUP98-HOXA9^+^ (bcCML only) and retro-orbitally transplanted into cohorts of sublethally irradiated (6 Gy) C57BL/6J mice. For AML-ETOa9 and NRAS, cells were transplanted into lethally irradiated (9.5 Gy) C57BL/6J recipients along with 3 × 10^5^ RBC lysed bone marrow rescue cells. For secondary transplants, Lin^−^ cells from primary bcCML recipient mice were transplanted into secondary sublethally irradiated recipients. The recipients were maintained on acid water HYDROPAC and irradiated sulfatrim diet and evaluated daily. Recipients receiving additional taurine (T8691, Sigma-Aldrich) were maintained on regular chow without taurine (D10012Gi, Research Diets). Taurine was dissolved in autoclaved acid water from the HYDROPAC and supplied in sterile water bottles. For in vivo venetoclax treatments, venetoclax (ABT-199; Tocris Bioscience) solution was made fresh daily in a solvent containing 10% ethanol with 60% Phosal 50 PG and 30% PEG-400, and delivered by oral gavage at a final dose of 50 mg per kg. GES (Toronto Research Chemicals or MedChemExpress) and TAG-HCl (synthesized by Enamine) were dissolved in autoclaved acid water from the HYDROPAC and supplied in sterile water bottles (GES) or intraperitoneally (i.p.) (TAG-HCl). For in vivo rapamycin treatments, rapamycin (Selleck Chemicals) stock solution (50 mg ml^−1^) was made in ethanol (Sigma-Aldrich). Single-use aliquots of the stock were diluted fresh each day in vehicle containing equal parts of 10% PEG-40 with 8% ethanol and 10% Tween-80 solutions. Mice received i.p. 5 mg per kg rapamycin or vehicle from days 5–10 after transplant. Premorbid animals were euthanized at the indicated experimental timepoints or at the end point. For all experiments, mice were monitored closely for signs of disease or morbidity daily and were euthanized after visible signs of hunched dorsum, failure to thrive or any signs of infection. These limits were not exceeded for any experiment. Relevant tissues were collected and analysed by flow cytometry, RNA-seq, proteomics, metabolomics or fixed for histology. Apoptosis assays were done using annexin V and 7AAD (eBiosciences). Analysis of in vivo bromodeoxyuridine (BrdU) incorporation was performed using the APC BrdU Flow Kit (BD Biosciences) after a single i.p. injection of BrdU (2 mg at 10 mg ml^−1^).

### RNA extraction and RT–qPCR

RNA was extracted using the RNeasy Micro or Mini kits (Qiagen) according to the manufacturer’s protocols. RNA concentrations were determined using NanoDrop 1000 Spectrophotometer (Thermo Fisher Scientific). RNA quality was assessed with the Agilent Bioanalyser 2100 (Agilent Technologies). Quantitative PCR with reverse transcription (RT–qPCR) was performed on the Bio-Rad CFX96 C100 Thermocycler using Bio-Rad CFX Manager 1.1 v.4.1 (Bio-Rad) or Thermo Fisher Scientific Quant Studio 12K Flex Real Time PCR using Quant Studio v.1.2 (Thermo Fisher Scientific). RT–qPCR data were analysed using Bio-Rad CFX Manager 1.1 v.4.1 or Quant Studio v.1.2.

### Isolation of mouse stromal cells for scRNA-seq and analysis

Microenvironmental populations were isolated as previously described^61^. In brief, bone and bone marrow were isolated from long bones and pelvis in 1× Media 199 (Gibco) with 2% FBS (GeminiBio). Bone marrow cells from 3–5 mice per timepoint were digested for 30 min in HBSS containing 2 mg ml^−1^ dispase II (Gibco), 1 mg ml^−1^ collagenase type IV (Sigma-Aldrich) and 20 ng ml^−1^ DNase type II (Sigma-Aldrich). Bone spicules were digested for 60 min in PBS supplemented with 2.5 mg ml^−1^ collagenase type I (Stem Cell Technologies) and 20% FBS. Digested bone marrow was red-blood-cell-lysed using RBC Lysis Buffer (eBioscience). Bone and bone marrow cells were pooled and CD45^+^TER119^+^ haematopoietic cells were magnetically depleted on the autoMACS cell separator (Miltenyi Biotec). The CD45^−^TER119^−^ stromal cells were either stained and analysed for candidate populations by flow cytometry (BD LSRFortessa) or further enriched by sorting (BD FACSAria II) and processed for scRNA-seq.

### Mouse bone marrow scRNA-seq and analysis

Cell suspensions were processed to generate scRNA-seq libraries using the Chromium Next GEM Single Cell 3′ GEM, Library and Gel Bead Kit v3.1 (10x Genomics) according to the manufacturer’s recommendations. The samples were loaded onto the Chromium Single-Cell Instrument (10x Genomics) to generate single-cell gel bead in emulsions (GEMs). GEM reverse transcription (GEM-RT) was performed to produce a barcoded, full-length cDNA from polyadenylated mRNA. After incubation, GEMs were broken, the pooled GEM-RT reaction mixtures were recovered and cDNA was purified with silane magnetic beads (DynaBeads MyOne Silane Beads, Thermo Fisher Scientific). The purified cDNA was further amplified by PCR to generate sufficient material for library construction. Enzymatic fragmentation and size selection was used to optimize the cDNA amplicon size and indexed sequencing libraries were constructed by end repair, A-tailing, adaptor ligation and PCR. The final libraries contained the P5 and P7 priming sites used in Illumina bridge amplification. Mouse samples were sequenced on the Illumina NovaSeq 6000 S2 flowcell while human samples were sequenced across several lanes of the Illumina NovaSeq X-plus 25B flowcell. The samples were demultiplexed and counted using cellranger v.4.0.0 mkfastq and count, using the default parameters. The samples were aligned to a custom reference containing the 10x provided mm10-2020-A mouse reference and an additional eGFP sequence.

Seurat v.4.1.0 within R v.4.1.1 was used for most of processing. Moreover, dplyr v.1.2.0 and tidyverse v.1.3.2 were used extensively for data piping and transformation. Samples were imported and cells were filtered for at least 200 features captured per cell and features were filtered for expression in at least 3 cells. Additional filters were applied, filtering out all cells with higher than 5% mitochondrial content and filtering out all cells positive for CD45, CD71, TER119 and eGFP. All samples were merged using ‘merge’ and normalized using SCTransform, regressing out the impact of mitochondrial features. Principal component analysis was performed using the first 40 principal components (RunPCA) and clusters were generated using FindNeighbors and FindClusters (resolution=0.5). UMAP dimensional reduction was also performed using RunUMAP using the first 30 principal components. Clusters were initially typed using scMCA v.0.2.0. Populations typed as non-stromal (B cell, pre-B cell, pro-erythrocyte, pro-erythroblast, neutrophil and megakaryocyte) were filtered from the dataset. Populations were further typed using published bone marrow stroma markers^13^. FindAllMarkers was used within the context of specific populations to determine which genes change significantly over time. DEGreport-implemented (v.1.30.3) degPatterns was used to tie lineage-specific expression patterns to the timepoints. Expression patterns corresponding to broadly increased or decreased expression over time were passed to gene set enrichment against KEGG_2019, GO_Biological_Process_2021, WikiPathways_2019_Mouse and ChEA_2022 databases using EnrichR-3.0.

### Human bone marrow microenvironment scRNA-seq analysis

Bone marrow aspirates were obtained from patients with MDS/AML after written informed consent in accordance with the Declaration of Helsinki and approval of University of Rochester institutional review board (IRB). To isolate the bone spicules, bone marrow aspirates were passed through a 40 μm cell strainer. The filter containing spicules was washed with PBS and placed in a six-well plate. The spicules were digested for 1 h in collagenase (Stem Cell Technologies) at 37 °C. After filtering, the filtered aspirate was used to isolate bone marrow mononuclear cells by density centrifugation (Ficoll-Paque, GE Healthcare). Digested bone cells and bone marrow mononuclear cells were pooled and stained with CD45–APC (BD Biosciences) followed by staining with anti-APC microbeads (Miltenyi Biotec). The stained cells were magnetically depleted for CD45^−^ cell fraction using LD columns (Miltenyi Biotec). The CD45^−^ cells were processed for scRNA-seq as described above.

Samples containing cells expressing COL1A1 were integrated into a single dataset using Seurat v.4.1.0 within R v.4.1.1. Cells with mitochondrial features making up greater than 25% of the detected transcripts were removed. The samples were scaled and normalized together, regressing out mitochondrial and globin-related content. Globin-related content was determined using HBB, HBA2, HBA1, HBD and HBM. Further regression was performed using Harmony v.0.1.0 to reduce the impact of sample specific effects. From there, the standard Seurat procedure was used, clustering to a resolution of 0.8.

A normal bone marrow reference was created using GEO GSE253355 (ref. ^23^). Data from this submission were normalized, PCA was run using the first 50 principal components, and UMAP was run using the first 50 principal components within Seurat v.5.0.3.9911. This dataset was then used within Azimuth v.0.5.0 to create the reference using Azimuth standard methods. The samples were then typed using this reference with the RunAzimuth function against the L1, L2 and coarse annotations.

### Primary human CD34^+^ cell RNA-seq analysis

Total RNA was purified with Qiagen RNeasy PLUS kit according to the manufacturer recommendations and eluted in nuclease-free water. The total RNA concentration was determined using the NanoDrop 1000 spectrophotometer (NanoDrop), and the RNA quality was assessed using the Agilent Bioanalyser (Agilent Technologies). The TruSeq Stranded mRNA Sample Preparation Kit (Illumina) was used for next generation sequencing library construction according to the manufacturer’s protocols. In brief, mRNA was purified from 200 ng total RNA with oligo-dT magnetic beads and fragmented. First-strand cDNA synthesis was performed with random hexamer priming followed by second-strand cDNA synthesis using dUTP incorporation for strand marking. End repair and 3′ adenylation was then performed on the double-stranded cDNA. Illumina adaptors were ligated to both ends of the cDNA, purified by gel electrophoresis and amplified with PCR primers specific to the adaptor sequences to generate cDNA amplicons of approximately 200–500 bp in size. The amplified libraries were hybridized to the Illumina single end flow cell and amplified using the cBot (Illumina). Single-end reads of 100 nucleotides were generated for each sample using Illumina’s HiSeq2500v4.

Raw reads generated from the Illumina basecalls were demultiplexed using bcl2fastq v.2.19.0. Quality filtering and adapter removal was performed using FastP v.0.20.1. Processed reads were then mapped to the human reference genome (hg38 + gencode v36; https://www.gencodegenes.org/human/release_36.html) using STAR_2.7.6a. Reads mapping to genes were counted using subread featurecounts v.2.0.1. Differential expression analysis was performed using DESeq2 v.1.28.1 with a *P*_adj_ threshold of 0.05 within R v.4.0.2. Gene Ontology analyses were performed using the EnrichR-3.0 package.

### Determining cell surface protein–ligand interactions

To determine significantly expressed receptors, the differential expression results from human RNA-seq described above were first filtered for significantly changing (*P*_adj_ < 0.05) genes with a log-transformed fold change value of greater than 0. A list of potential cell surface receptors was generated using this gene list in conjunction with cell surface proteins detailed within the Cell Surface Protein Atlas^19^ and essential for LSC growth in vivo^6^. The resultant gene list was further reviewed for genes that are not truly expressed within the cell surface, leading to the removal of CHST11, ST3GAL4, ACAA1, CLCN6, CHPF2, CTSK, ATP6AP1, CTSD, PNPLA6, DMXL2, TUBB6, MAN2B2, CLN3, MGAT4B, MYH9 and PIGG from further review. Ligand expression from the temporal scRNA-seq dataset corresponding to bcCML and AML expressed receptors was determined by differential expression across the populations. Differentially expressed genes were filtered for corresponding ligands using a combination of nichenetr (v.1.1.0)^62^ and literature-supported interactions. These included NRP1^63^, ADSL^64,65^, CDO1 and CSAD for taurine synthesis^66^, GADL1 and CNDP1 for β-alanine synthesis^67,68^, KIT^69^, CD155^70^ and CD33^71^ for those not captured within this mapping. The Broad Institute hosted dataset^20^ was used as a stand in for healthy immune microenvironment within the context of AML. Counts (RNA_soupX1.mtx.gz) and metadata (metadata_clustering_w_header_upd.csv) were downloaded from the Broad Single Cell Portal (https://singlecell.broadinstitute.org/single_cell/study/SCP1987/an-inflammatory-state-remodels-the-immune-microenvironment-and-improves-risk-stratification-in-acute-myeloid-leukaemia) and imported using Seurat. FindMarkers was used to determine which genes were upregulated within the microenvironment in relation to the other populations. Significantly expressed genes were then filtered for ligands using the same method as the stromal microenvironment. Significantly expressed ligand and receptor mappings within the stroma microenvironment, the immune microenvironment and within bcCML and AML human bulk RNA-seq data were visualized using circlize v.0.4.15. For illustration purposes, ligands expressed with a log-transformed fold change of 5 or higher were limited to 5.

### Mouse *Slc6a6* wild-type and knockout leukaemia RNA-seq

The RNeasy Plus Micro Kit (Qiagen) was used for RNA extraction. RNA concentration was determined using the NanoDrop 1000 spectrophotometer (NanoDrop), and the RNA quality was assessed using the Agilent Bioanalyser 2100 (Agilent Technologies). A total of 1 ng of total RNA was pre-amplified using the SMARTer Ultra Low Input kit v4 (Clontech) according to the manufacturer’s recommendations. The quantity and quality of the subsequent cDNA was determined using the Qubit Fluorometer (Life Technologies) and the Agilent Bioanalyser 2100 (Agilent). Then, 150 pg of cDNA was used to generate Illumina-compatible sequencing libraries using the NexteraXT library preparation kit (Illumina) according to the manufacturer’s protocols. The amplified libraries were hybridized to the Illumina flow cell and sequenced using the Illumina NextSeq 550 (Illumina). Single-end reads of 100 nucleotides were generated for each sample. The mouse bulk RNA-seq samples were processed otherwise identical to the human bulk RNA-seq with two exceptions: m38 + gencode M27 reference (https://www.gencodegenes.org/mouse/release_M27.html) for use within alignment and counting, and ‘-s 0’ being used within subread featureCounts. See the ‘Primary human CD34^+^ cell RNA-seq analysis’ section for details on differential expression analysis.

### Primary human patient-derived cells and human leukaemia cell lines

For human RNA-seq experiments and other studies, CD34^+^ cells were isolated from bone marrow samples of healthy donors and samples from patients with AML and bcCML obtained under University of Rochester institutional review board-approved protocols with written informed consent in accordance with the Declaration of Helsinki. The normal human CD34^+^ HSPCs used in all functional assays were purchased (Stem Cell Technologies). Cells were cultured in Iscove’s modified Dulbecco’s medium with 10% FBS, 100 IU ml^−1^ penicillin–streptomycin (Gibco) and 55 μM 2-mercaptoethanol, 1× LDL (Sigma-Aldrich) and supplemented l-glutamine with 100 ng ml^−1^ SCF and TPO (R&D Systems). Human leukaemia cell lines K562, THP1 and M-V-411 (ATCC) were maintained in RPMI/IMDM with 10% FBS, 100 IU ml^−1^ penicillin–streptomycin (Gibco). These cell lines were validated by vendor. MDS-L cells (from K. Tohyama) were authenticated in house by flow cytometry as CD45^+^CD34^+^CD38^+^ and maintained in RPMI with 10% FBS supplemented with 20 ng ml^−1^ IL-3 (PeproTech). Cell lines were not tested for mycoplasma. For colony-forming assays with shRNAs, leukaemia and normal cells were transduced with the indicated lentiviral shRNAs. Cells were sorted 24 h after infection and plated in CFU assays or transplanted in sublethally irradiated NSG mice.

### MSC isolation, osteogenic differentiation and co-culture with leukaemia cells

MSCs were isolated from leukaemic mice and cultured in 10 cm dishes in MEMα with no ascorbic acid (Gibco) supplemented with 15% FBS and 100 IU ml^−1^ penicillin–streptomycin (Gibco). Then, 6 days after culture initiation, the cells were sorted for CD45^−^CD3^−^B220^−^TER119^−^GR1^−^CD31^−^CD51^+^SCA1^+^ MSCs. Sorted cells were expanded in the medium descried above. For co-culture experiments, 50,000 MSCs were plated in a 48-well plate, and transduced with the indicated lentiviral shRNAs. Then, 3 days after infection, osteogenic differentiation was induced by switching to MEMα (Gibco) supplemented 15% FBS, 100 IU ml^−1^ penicillin–streptomycin (Gibco) along with 50 μg ml^−1^ ascorbic acid (Sigma-Aldrich), 10 mM β-glycerolphosphate (Sigma-Aldrich) and 100 nM dexamethasone (Sigma-Aldrich) for 6 days. On day 6 after differentiation, 100,000 Lin^−^ bcCML cells were added in X-Vivo supplemented with 10% FBS, 50 μM 2-mercatpoethanol and penicillin–streptomycin. Then, 3 days after co-culture, leukaemia cells were analysed for cell viability and plated in methylcellulose for colony-forming assays. Microscopy images were obtained on THE Olympus CKX41 SYSTEM using CellSens Entry v.2.3 (Olympus).

### Normal HSC in vivo transplantation assays

For bone marrow transplants, 500 HSCs were isolated from bone marrow of *Slc6a6*^*+/+*^ or *Slc6a6*^*−/−*^ mice and transplanted into lethally irradiated (9.5 Gy) CD45.1 mice along with 2 × 10^5^ SCA1-depleted bone marrow rescue cells. For subsequent secondary transplants, 2 × 10^6^ red-blood-cell-lysed bone marrow cells isolated from primary recipient mice were transplanted into lethally irradiated (9.5 Gy) CD45.1 mice. Peripheral blood of recipient mice was collected every 4 weeks for 4 months after transplant and bone marrow analysed at the end of 4 months.

### Methylcellulose colony-formation assays

For colony assays with mouse cells, the indicated numbers of Lin^−^ bcCML cells or KIT^+^ AML cells were plated in methylcellulose medium (M3234, StemCell Technologies). Colonies were scored at 7 days. For colony assays with human cell lines or patient-derived AML samples, cells were plated in methylcellulose medium (H4434; StemCell Technologies). Colony numbers were counted 10–14 days after plating. Taurine antagonist or TAG (synthesized by Enamine; 92% pure), GES (G827500, Toronto Research Chemicals), taurine (T8691, Sigma-Aldrich), mTOR activator (MHY1485, Sigma-Aldrich) and Venetoclax (ABT-199, Tocris Bioscience) were used as described. Venetoclax and GES Synergy quantification was calculated using Chou-Talalay method^72^.

### Seahorse assays

The Seahorse XF Glycolysis Stress Test Kit (Agilent Technologies, 103020-100) and the Seahorse XF cell mito stress test kit (Agilent Technologies, 103015-100) were used to measure glycolytic flux (ECAR) and oxygen consumption (OCR) respectively. Mouse Lin^−^ bcCML cells were sorted and cultured for 48 h in X-Vivo supplemented with 10% FBS, SCF and TPO. Then, 1 h before the analysis, 50,000–100,000 cells were seeded in Cell-Tak-coated (Corning, 324240) 96-well XF96 well plates in Seahorse XF medium (Agilent Technologies, 102353-100), and the plate was incubated at 37 °C. ECAR data were measured after sequential addition of glucose (10 mM), oligomycin (1μM) and 2-deoxyglucose (50 mM) using the XF96 analyser (Agilent Technologies). OCR data were measured after sequential addition of oligomycin (1.5 μM), carbonyl cyanide-4 (trifluoromethoxy) phenylhydrazone (FCCP) (0.5 μM), rotenone and antimycin (0.5 μM) using the XF96 analyser. Data were analysed using Wave v.2.6.3 (Agilent Technologies).

### Western blot analysis

Cell lysates prepared in 1× RIPA (Thermo Fisher Scientific) supplemented with 1× protease, 1× phosphatase inhibitors (Cell Signaling Technology) and 250 IU benzonase nuclease (Millipore Sigma) were separated on gradient polyacrylamide gels and transferred to nitrocellulose blotting membrane (0.45 μM; GE Healthcare). Primary antibodies against phosphorylated mTOR, mTOR, phosphorylated S6K, S6K, pEIF4B, EIF4B and β-actin (Cell Signaling Technology) or tubulin (Abcam) were used. Horse-radish peroxidase (HRP)-conjugated anti-rabbit antibodies (Cell Signaling Technology) were used to detect primary antibodies. Immunoblots were developed using SuperSignal West Femto Maximum Sensitivity Substrate (Thermo Fisher Scientific). Immunoblots were imaged using the LI-COR Odyssey M system using Empiria Studio v.2.3 (LI-COR). Images were analysed using Empiria Studio v.3.2.0.186 (LI-COR). Raw gel images are provided in Supplementary Fig. 1.

### Protein extraction, sample preparation and MS analysis

#### Protein extraction

Cells were lysed in 50 μl of 5% SDS, 100 mM triethylammonium bicarbonate (TEAB, Thermo Fisher Scientific) and 50 μg of protein from each sample was reduced with dithiothreitol (Sigma-Aldrich). Proteins were alkylated and trapped to S-Trap micros (Protifi), digested with trypsin and extracted by sequential additions of 0.1% trifluoroacetic acid (TFA) in acetonitrile. Then, 1% of each sample was used for global DIA analysis, and the remaining sample was frozen and dried down in the Speed Vac (Labconco) before TMT labelling. The samples were reconstituted in TEAB and labelled with TMT 10-plex reagents (Thermo Fisher Scientific) according to the manufacturer’s protocol. All of the samples were combined and dried down in a speed vac before desalting with 0.1% TFA in acetonitrile using the 130 mg C18 sorbent sep-pak attached to a 3 ml syringe (Waters). Desalted samples were frozen and dried down before phosphorylation enrichment using the High-Select FeNTA Enrichment Kit (Thermo Fisher Scientific) according to the manufacturer’s protocol. Enriched phosphorylated samples were frozen, dried down and fractionated using C18 spin columns. The fractions were eluted by stepwise addition of 10 mM AmmOH with increasing acetonitrile concentrations: 3.5, 6.5, 9.5, 12.5, 15.5, 18.5, 27, 50%. The eight fractions were concatenated to four by combining fractions 1/5, 2/6, 3/7, 4/8. Fractionated samples were frozen, dried down and reconstituted in 0.1% TFA for MS analysis.

#### MS analysis

Non-TMT-tagged peptides were injected onto a 75 μm × 2 cm trap column before refocusing on a 100 μm × 15 cm C18 column with 1.8 μm beads (Sepax) using the Vanquish Neo UHPLC (Thermo Fisher Scientific) system connected to the Orbitrap Astral mass spectrometer (Thermo Fisher Scientific). Ions were introduced to the mass spectrometer using a Nanospray Flex source operating at 2 kV. Solvent A (0.1% formic acid in water) and solvent B (0.1% formic acid in 80% acetonitrile) formed the gradient starting at 1% B and ramped to 99% B for a total runtime of 14 m. After each run was completed, the column was re-equilibrated with 1% B before the next injection. The Orbitrap Astral was operated in data-independent acquisition (DIA) mode for global proteomic analysis, with MS1 scans acquired at a resolution of 240,000 with a maximum injection time of 5 ms over a range of 390–980 *m*/*z*. DIA MS2 scans were acquired using 2 Da windows, a 3 ms maximum injection time, an HCD collision energy of 25% and a normalized automatic gain control (AGC) of 500%. Fragment ions were collected over a scan range of 150–2,000 *m*/*z*. Phosphoprotein analysis was carried out on the same Vanquish Neo UHPLC and Orbitrap Astral mass spectrometer system, but with several changes to the LC and instrument settings. To account for ratio compression inherent with TMT, samples were high-pH fractionated to reduce complexity before MS analysis. A longer gradient of 5–30% B over 48 min was used to further separate phosphopeptides. A data-dependent acquisition method using a FAIMS Pro Duo (Thermo Fisher Scientific) was used with three compensation voltages (−40 V, −60 V, −80 V) to further reduce the sample complexity. For each CV, a full scan was acquired over a range of 400–1,500 *m*/*z* in the Orbitrap, while MS2 scans were analysed in the Astral analyser for 1 s, after which the instrument switched to the next CV and the process was repeated for a total cycle time of 3 s. Peptides with a charge state of between 2 and 6 were isolated based on intensity with a 0.5 Da isolation window, and were fragmented with an HCD collision energy of 35%. The maximum injection time was 20 ms, and the normalized AGC target was 100%. Dynamic exclusion was set to 15 s.

#### Data analysis

The global DIA raw data were processed with DIA-NN v.1.8.1 (https://github.com/vdemichev/DiaNN) using library-free analysis mode. The library was annotated using the *Mus musculus* UniProt database (UP000005640_9606). For precursor ion generation, the maximum number of missed cleavages was set to 1, maximum number of variable modifications to 1 for Ox(M), peptide length range to 7–30, precursor charge range to 2–3, precursor *m*/*z* range to 380–980, and fragment *m*/*z* range to 150–2,000. The quantification was set to ‘Robust LC (high precision)’ mode with RT-dependent median-based cross-run normalization enabled, MBR enabled, protein inferences set to ‘Genes’ and ‘Heuristic protein inference’ turned off. Precursors were filtered at library precursor *q*-value (1%), library protein group *q*-value (1%) and posterior error probability (50%). Protein quantification was carried out using the MaxLFQ algorithm (https://github.com/vdemichev/diann-rpackage) and the number of peptides quantified in each protein group was determined using the DiannReportGenerator Package (https://github.com/URMC-MSRL/DiannReportGenerator). Further filtering, missing value imputation and statistical tests were performed using Perseus^73^. Phosphoproteome raw data were searched using the CHIMERYS within the Proteome Discoverer software platform v.3.1 (Thermo Fisher Scientific), allowing for up to two missed cleavages, with a fragment mass tolerance of 10 ppm. Carbamidomethyl on cysteine, and TMT on lysine and peptide N terminus were set as fixed modifications, while oxidation of methionine and phosphorylation of serine, threonine and tyrosine were set as variable modifications. Reporter ions were quantified using the Reporter Ions Quantifier node, with an integration tolerance of 20 ppm, and the integration method was set to ‘most confident centroid’.

### Immunohistochemical staining and analysis

Paraffin-embedded 4 μm human bone marrow biopsy sections were deparaffinized in xylene and antigen epitopes were retrieved using BOND epitope retrieval solution (pH 9) for 10 min. Endogenous peroxidases were quenched with a 10 min incubation in 3% H_2_O_2_-methanol solution. The sections were then blocked in 10% donkey serum for 60 min. The sections were either incubated with anti-CDO1 (Proteintech) overnight or with anti-osterix (Abcam) for 2 h at room-temperature. The sections were washed in PBS and stained with HRP-conjugated donkey anti-rabbit secondary antibody (Jackson Immunoresearch) for 1.5 h at room temperature. After three washes, colour was developed using the ImmPACT DAB Substrate kit (Vector laboratories, SK-4105) according to the manufacturer’s protocol. The sections were then counterstained with haematoxylin. Three different areas of each section were imaged on the Olympus BX41 microscope with a ×20 (0.5 NA) objective. Images were analysed using the IHC plugin toolbox in Fiji v.1.54g.

### Immunofluorescence staining

Leukaemia cells were cultured in RPMI without amino acids (US Biologicals) in the presence or absence of taurine (Sigma-Aldrich) and seeded on eight-well cover glass chambers (BD biosciences) coated with Cell-Taq (Corning) according to the manufacturer’s protocols. MSCs were grown and differentiated in 35 mm glass-bottom dishes with 14 mm microwell (MatTek Life Sciences). Cells were fixed with 4% PFA and blocked in blocking buffer (PBS with 5% donkey serum, 1% BSA and 0.1% Triton X-100) and incubated overnight in primary antibodies diluted in the blocking buffer. Primary antibodies used included mTOR (Cell Signaling Technologies), LAMP1 (DHSB) or CDO1 (Proteintech). Cells were washed in PBS containing 0.1% Tween-20 (Sigma-Aldrich), stained with Alexa-Fluor-conjugated secondary antibodies (Thermo Fisher Scientific), and mounted in Fluormount G (Thermo Fisher Scientific).

### Immunofluorescence imaging and analysis

Immunofluorescence images were acquired with the Teledyne Photometrics Prime BSI express sCMOS camera mounted on the Nikon ECLIPSE Ti2 inverted microscope equipped with the NIS-Elements 6D imaging acquisition module (v.5.42.06). The Nikon D-LEDI fluorescence LED illumination system (equipped with 385 nm, 488 nm, 568 nm and 621 nm excitation wavelengths) was used as the primary illumination source. Specific illumination wavelengths were selected by combining a large field of view quad-filter cube (DAPI/FITC/TRITC/CY5; 96378) with specific Lumencor emission filters (FF01-474/27-32, FF01-515/30-32, FF01-595/31-32, FF02-641/75-32). MSC and osteolineage cultures were imaged with the Nikon CFI60 Plan Apochromat Lambda D ×20 (0.8 NA) objective lens, and leukaemia cells were imaged using the Nikon CFI60 Plan Apochromat Lambda D ×100 (1.45 NA) objective lens. Images were deconvoluted using Imaris v.10.2 (Oxford instruments), and Pearson’s co-localization analysis was performed using the JACoP BIOP plugin in Fiji v.1.54g.

### Phosphoflow cytometry

Lineage depleted *Slc6a6*^*+/+*^ and *Slc6a6*^*−/−*^ mouse leukaemia cells were fixed using BD Cytofix/Cytoperm Fixation/Permeabilization Kit (BD Bioscience) according to the manufacturer’s protocols. Cells were stained with primary antibodies against phosphorylated mTOR (Cell Signaling Technology). Cells were then stained with donkey anti-rabbit secondary antibody conjugated with Alexa Fluro 488 (Invitrogen) to detect p-mTOR. Analysis was performed on the LSRFortessa (BD Biosciences) system. Data were analysed using FlowJo software.

### Taurine quantification

Bone marrow cells or bone marrow peripheral fluid were isolated from femurs in Hanks’ balanced salt solution (Gibco) with 5% FBS and 2 mM EDTA. For taurine analysis of bone marrow cells after genetic loss of *Slc6a6* or treatment of leukaemia cells with taurine inhibitor treatments, cells were lysed in RIPA buffer (Thermo Fisher Scientific) with benzonase nuclease (Sigma-Aldrich). For bone marrow interstitial fluid analysis, one femur was crushed in 1 ml of buffer, filtered and centrifuged at 1,500*g* for 5 min. The supernatant was concentrated using 10,000 MWCO spin columns (Corning). Then, 25 μl of concentrated samples was quantified using the Taurine Assay kit (Sigma-Aldrich/Abcam) according to the manufacturer’s protocols on the BioTek Synergy 2 plate reader using Gen5 v.3.11 (BioTek). The samples were corrected for taurine amounts in unconditioned fresh medium or buffer. Alternatively, cell pellets were processed as described in the liquid chromatography–mass spectrometry (LC–MS) section below, and taurine levels were measured by LC–MS (Orbitrap Exploris 240).

### Untargeted metabolomics of Lin^−^ leukaemia cells

For untargeted metabolomics, spleens from mice bearing *Slc6a6*^+/+^ or *Slc6a6*^*−/−*^ leukaemias were quickly dissected and dissociated in Hanks’ balanced salt solution (Gibco) with 5% FBS and 2 mM EDTA at 4 °C. Leukaemia cells were collected and maintained at 4 °C all through the process of isolation, staining and magnetic sorting to minimize metabolic changes. Lin^+^ (CD3ε^−^CD4^−^CD8^−^GR1^−^CD11b^−^TER119^−^CD45R^−^CD19^−^) leukaemia cells were magnetically depleted using LD columns (Milteny Biotec). Lin^−^ leukaemia stem cell fractions were washed with PBS containing 5 mM glucose and centrifuged at 3,000*g* for 1 min, snap-frozen and processed for metabolomics as described in the ‘LC–MS analysis’ section below.

### ^13^C-taurine tracing in leukaemia cells

K562 cells were cultured in serum-free RPMI-1640 medium in six-well plates for 48 h. The cells were then cultured in serum free RPMI-1640 supplemented with 200 μM taurine (1,2 ^13^C_2_, 98%; Cambridge Isotope Labs) or no additional taurine for 24 h. The cells were washed with PBS containing 5 mM glucose and centrifuged at 3,000*g*, snap-frozen and processed for metabolomics as described below in the ‘LC–MS analysis’ section.

### LC–MS analysis

Frozen cell pellets were resuspended at 2 million cells per 1 ml of 80% methanol by vortexing, transferred to −80 °C for 30 min and then to regular ice for 30 min with vortexing every 10 min. Next, the samples were centrifuged at 17,000*g* for 10 min and 90% of supernatant was dried down in a vacuum evaporator (Thermo Fisher Scientific). The samples were reconstituted in 50% acetonitrile (A955, Thermo Fisher Scientific) at a volume equal to 10% of the dried down volume and transferred to glass vials for LC–MS analysis. The metabolite extracts were analysed by high-resolution MS with the Orbitrap Exploris 240 (Thermo Fisher Scientific) system coupled to the Vanquish Flex LC system (Thermo Fisher Scientific). Then, 2 µl of the samples was injected onto the Waters XBridge XP BEH Amide column (150 mm length × 2.1 mm inner diameter, 2.5 µm particle size) maintained at 25 °C, with a Waters XBridge XP VanGuard BEH Amide (5 mm × 2.1 mm inner diameter, 2.5 µm particle size) guard column. For positive-mode acquisition, mobile phase A was 100% LC–MS-grade H_2_O with 10 mM ammonium formate and 0.125% formic acid. Mobile phase B was 90% acetonitrile with 10 mM ammonium formate and 0.125% formic acid. For negative-mode acquisition, mobile phase A was 100% LC–MS-grade H_2_O with 10 mM ammonium acetate, 0.1% ammonium hydroxide and 0.1% medronic acid (Agilent). Mobile phase B was 90% acetonitrile with 10 mM ammonium acetate, 0.1% ammonium hydroxide and 0.1% medronic acid. The gradient was 0 min, 100% B; 2 min, 100% B; 3 min, 90% B; 5 min, 90% B; 6 min, 85% B; 7 min, 85% B; 8 min, 75% B; 9 min, 75% B; 10 min, 55% B; 12 min, 55% B; 13 min, 35% B; 20 min, 35% B; 20.1 min, 35% B; 20.6 min, 100% B; 22.2 min, 100% B; all at a flow rate of 150 μl min^−1^, followed by 22.7 min, 100% B; 27.9 min, 100% B at a flow rate of 300 μl min^−1^, and finally 28 min, 100% B at flow rate of 150 μl min^−1^, for a total length of 28 min. The H-ESI source was operated in positive mode at spray voltage 3,500 V or negative mode at spray voltage 2,500 V with the following parameters: sheath gas 35 au, aux gas 7 au, sweep gas 0 au, ion transfer tube temperature 320 °C, vaporizer temperature 275 °C, mass range 70 to 1,000 *m*/*z*, full scan MS1 mass resolution of 120,000 FWHM, RF lens at 70% and standard AGC. LC–MS data were analysed using Compound Discover (v.3.3, Thermo Fisher Scientific) and El-Maven software^74^ for peak-area determination and compound identification. Compounds were identified by matching to LC–MS method-specific retention time values of external standards and MS^2^ spectral matching to external standards and the mzCloud database (Thermo Fisher Scientific). Raw *P* values were calculated using pairwise Mann–Whitney–Wilcoxon rank-sum tests and *P*_adj_ values were computed using Benjamini–Hochberg false-discovery rate correction. Data were uploaded to the Metabolomics Workbench^75^.

### Statistical analysis

Statistical analyses were performed using GraphPad Prism software v.6.0 (GraphPad). Data are mean ± s.e.m. One-way ANOVA, two-way ANOVA, unpaired two-sided Student’s *t*-tests, multiple unpaired *t*-tests corrected with the Benjamin–Hochberg method, ratio-paired *t*-tests, and log-rank tests were used to determine statistical significance. Combination index and isobologram plots were made using CompuSyn^72^.

### Reporting summary

Further information on research design is available in the Nature Portfolio Reporting Summary linked to this article.

## Online content

Any methods, additional references, Nature Portfolio reporting summaries, source data, extended data, supplementary information, acknowledgements, peer review information; details of author contributions and competing interests; and statements of data and code availability are available at 10.1038/s41586-025-09018-7.

## Supplementary information

Supplementary Figure 1 Uncropped western blots.

Reporting Summary

Supplementary Table 1 List of shRNA and primer sequences.

Supplementary Table 2 List of antibodies used.

## Source data

Source Data Fig. 1

Source Data Fig. 2

Source Data Fig. 3

Source Data Fig. 4

Source Data Fig. 5

Source Data Extended Data Fig. 1

Source Data Extended Data Fig. 4

Source Data Extended Data Fig. 5

Source Data Extended Data Fig. 6

Source Data Extended Data Fig. 7

Source Data Extended Data Fig. 8

Source Data Extended Data Fig. 9

Source Data Extended Data Fig. 10

Source Data Extended Data Fig. 11

Source Data Extended Data Fig. 12

## Data Availability

The scRNA-seq and bulk RNA-seq data for this publication are available under GEO accessions GSE226372 (human bulk RNA-seq), GSE227082 (mouse bulk RNA-seq), GSE226644 (mouse temporal scRNA-seq) and GSE288862 (scRNA-seq of human MDS and AML bone marrow microenvironment). A shiny app hosting the mouse temporal scRNA-seq is available online (https://wilmot-genomics.shinyapps.io/gse226644/). Receptor and ligand interactions were determined using the NicheNet^62^, Cell Surface Protein atlas^19^, AML and healthy immune microenvironment^20^ datasets. The link between *SLC6A6* and *LDLR* expression and AML prognosis was determined using UCSC Xena Browser (https://xenabrowser.net/). Wild-type and *Slc6a6*^*−/−*^ proteomics data are available at ProteomeXchange (PXD062322). The library was annotated using *M. musculus* UniProt ‘one protein sequence per gene’ database (UP000005640_9606, downloaded April 2021). WT and *Slc6a6*^*−/−*^ metabolomics data (ST003835) and ^13^C-taurine tracing data (ST003836) are available online. Source data are provided with this paper.

## Code availability

Mouse and human scRNA datasets were processed using Seurat (v.4.1.0; https://github.com/satijalab/seurat/releases/tag/v4.1.0) within R (v.4.1.1; https://github.com/r-hub/R/releases/tag/v4.1.1). Time-course analysis was performed using DEGreport (v.1.30.3; https://lpantano.github.io/DEGreport/index.html). Pathway enrichment was performed using EnrichR (v.3.0; https://cran.r-project.org/web/packages/enrichR/). Sample integration within human scRNA datasets was performed using Harmony (v.0.1.0; https://github.com/immunogenomics/harmony). Integrating external datasets into the human scRNA dataset was done using Seurat (v.5.0.3.99911; https://github.com/satijalab/seurat/releases/tag/v5.0.3) and Azimuth (v.0.5.0; https://github.com/satijalab/azimuth) within R (v.4.3.1; https://github.com/r-hub/R/releases/tag/v4.3.1). Mouse and human bulk RNA datasets were trimmed and quality-filtered using FastP (v.0.20.1; https://github.com/OpenGene/fastp/releases/tag/v0.20.1). Read data were aligned using STAR (v.2.7.6a; https://github.com/alexdobin/STAR/releases/tag/2.7.6a) and counted using subread-featureCounts (v.2.0.1; https://subread.sourceforge.net/). Differential expression analysis was performed using DESeq2 (v.1.28.1; https://bioconductor.org/packages/release/bioc/html/DESeq2.html) within R (v.4.0.2; https://github.com/r-hub/R/releases/tag/v4.0.2). Pathway enrichment analysis within the bulk RNA datasets was performed using EnrichR (v.3.0, above). Ligand–receptor visualization was performed using NicheNetR (v.1.1.0; https://github.com/saeyslab/nichenetr) and Circlize (v.0.4.15; https://github.com/jokergoo/circlize) within R (v.4.1.1, above). Ggplot2 (https://github.com/tidyverse/ggplot2) and dplyr (https://github.com/tidyverse/dplyr) were used for figure generation and data manipulation throughout. A shiny app hosting the mouse temporal scRNA-seq is available online (https://wilmot-genomics.shinyapps.io/gse226644/) and was generated using ShinyCell (v.2.1; https://github.com/SGDDNB/ShinyCell).
