# Supplementary material for: Taurine from tumour niche drives glycolysis to promote leukaemogenesis
Source: Nature. 2025 May 14;644(8075):263–72. doi: 10.1038/s41586-025-09018-7 (PMC12328231; doi:10.1038/s41586-025-09018-7)

nature portfolio

https://doi.org/10.1038/s41586-025-09018-7

Supplementary information

Taurine from tumour niche drives glycolysis to promote leukaemogenesis

In the format provided by the authors and unedited

Supplementary Figure 1: Raw Data (Gels)

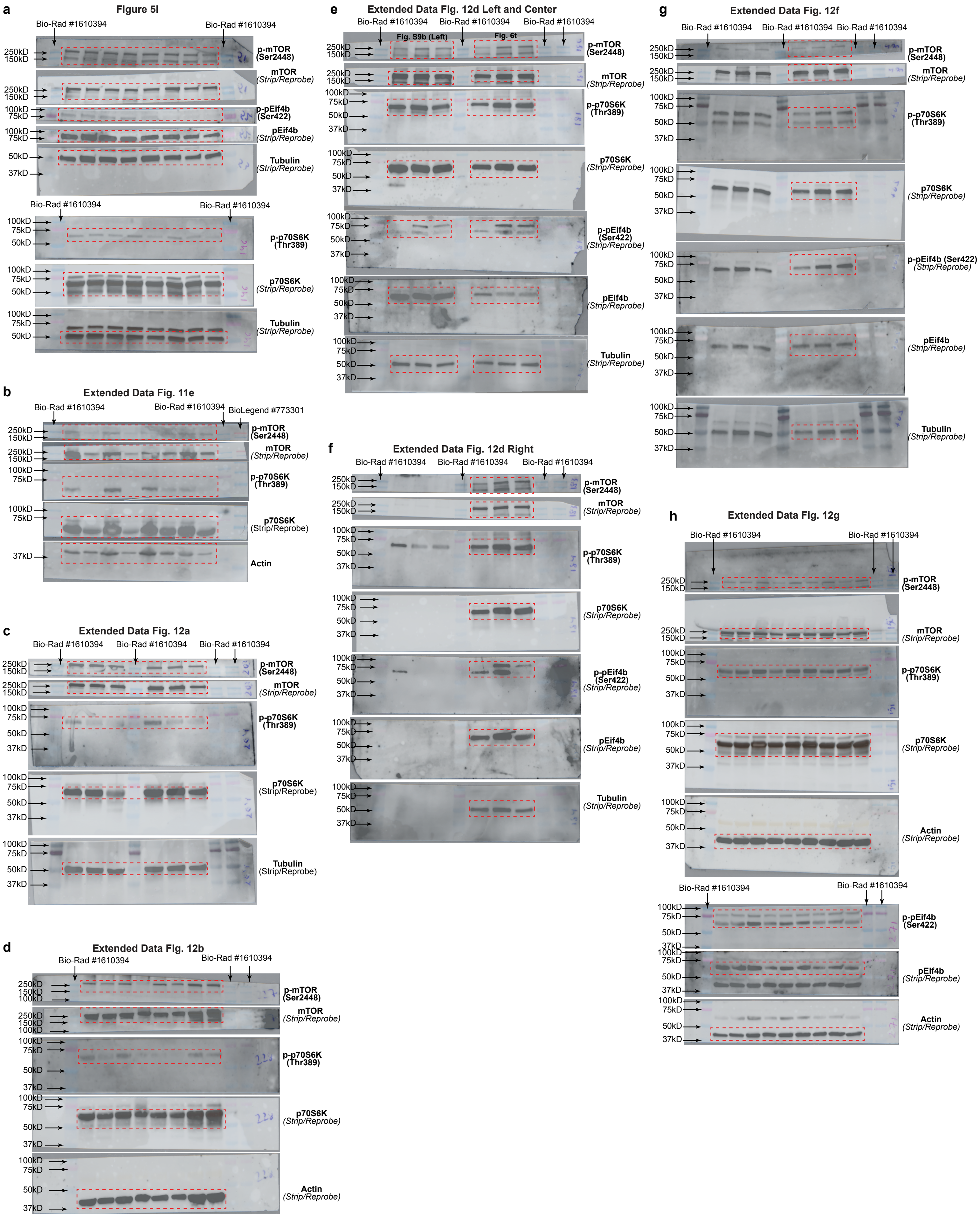

Supplement: Supplementary file 1 — Uncropped western blots. [file 41586_2025_9018_MOESM1_ESM.pdf]
