# Supplementary material for: Taurine from tumour niche drives glycolysis to promote leukaemogenesis
Source: Nature. 2025 May 14;644(8075):263–72. doi: 10.1038/s41586-025-09018-7 (PMC12328231; doi:10.1038/s41586-025-09018-7)
Supplement: Supplementary file 2 — Reporting Summary [file 41586_2025_9018_MOESM2_ESM.pdf]

nature portfolio

Corresponding author(s): Jeevisha Bajaj

Last updated by author(s): Apr 5, 2025

Reporting Summary

Nature Portfolio wishes to improve the reproducibility of the work that we publish. This form provides structure for consistency and transparency in reporting. For further information on Nature Portfolio policies, see our Editorial Policies and the Editorial Policy Checklist.

Statistics

For all statistical analyses, confirm that the following items are present in the figure legend, table legend, main text, or Methods section.

n/a	Confirmed	
☐	☒	The exact sample size (n) for each experimental group/condition, given as a discrete number and unit of measurement
☐	☒	A statement on whether measurements were taken from distinct samples or whether the same sample was measured repeatedly
☐	☒	The statistical test(s) used AND whether they are one- or two-sided<br>*Only common tests should be described solely by name; describe more complex techniques in the Methods section.*
☒	☐	A description of all covariates tested
☐	☒	A description of any assumptions or corrections, such as tests of normality and adjustment for multiple comparisons
☐	☒	A full description of the statistical parameters including central tendency (e.g. means) or other basic estimates (e.g. regression coefficient) AND variation (e.g. standard deviation) or associated estimates of uncertainty (e.g. confidence intervals)
☐	☒	For null hypothesis testing, the test statistic (e.g. F, t, r) with confidence intervals, effect sizes, degrees of freedom and P value noted<br>*Give P values as exact values whenever suitable.*
☒	☐	For Bayesian analysis, information on the choice of priors and Markov chain Monte Carlo settings
☒	☐	For hierarchical and complex designs, identification of the appropriate level for tests and full reporting of outcomes
☐	☒	Estimates of effect sizes (e.g. Cohen's d, Pearson's r), indicating how they were calculated

Our web collection on statistics for biologists contains articles on many of the points above.

Software and code

Policy information about availability of computer code

Data collection

Flow cytometry-based analysis was performed on LSR Fortessa and cell sorting was carried out on BD FACS Aria II using FACSDiva v9.0 (BD Biosciences).
qRT-PCR was carried out on BioRad CFX96 C100 Thermocycler using BioRad CFX Manager 1.1 v4.1 (BioRad) and Thermofisher Scientific Quant Studio 12K Flex Real Time PCR using using Quant Studio v1.2 (Thermofisher Scientific).
RNA and DNA concentrations were determined using the NanoDrop 1000 Spectrophotometer (Thermofisher Scientific). RNA quality was assessed with the Agilent Bioanalyzer 2100 (Agilent Technologies)
Western blots were performed using LI-COR Odyssey M using Empiria Studio v2.3 (LI-COR).
Taurine assay absorbance was measured using BioTek Synergy 2 plate reader using Gen5 v.3.11 (BioTek).
Seahorse assays were carried out on Agilent Technologies XF96 Analyzer using Wave v2.6.3 (Agilent Technologies).
Microscopy images were obtained on Olympus CKX41 using CellSens Entry v2.3 (Olympus).
Immunohistochemical images were obtained with Olympus BX41 and deconvolution was completed with Imaris v10.2 .
Immunoflurescence images were obtained with with a Teledyne Photometrics Prime BSI Express sCMOS Camera mounted on a Nikon ECLIPSE Ti2 inverted microscope equipped with the NIS-Elements 6D Imaging Acquisition Module (v5.42.06)
scRNA-Sequencing data was generated using NovaSeq 6000 (Illumina) and samples were counted using cellranger-4.0.0.
Bulk RNA Sequencing data was generated using Illumina HiSeq2500 v4 or llumina NextSeq 550.
Mass Spectrometry data was obtained Vanquish Neo UHPLC (Thermofisher Scientific) connected to Orbitrap Astral Mass Spectrometer (Thermofisher Scientific) . Data-dependent acquisition method utilized a FAIMS Pro Duo (Thermofisher Scientific).
LC/MS data was analyzed by high resolution mass spectrometry with an Orbitrap Exploris 240 (Thermofisher Scientific) coupled to a Vanquish Flex liquid chromatography system (Thermofisher Scientific).

Data analysis

Statistical analyses were performed using GraphPad Prism v9 and v10.
Flow cytometry data was analyzed using FlowJo software v10.9.0.
Seahorse data was analyzed using Wave V2.6.3 (Agilent Technologies)
qRT-PCR data was analyzed using BioRad CFX Manager 1.1 v4.1 and Quant Studio v1.2 (Thermofisher Scientific)
Western blot images were analyzed using Empiria Studio v3.2.0.186 (LI-COR)
Immunohistochemical images were analyzed using IHC plugin toolbox in Fiji v1.54g.
Immunioflurescence Images were deconvoluted using Imaris (Oxford instruments), and Pearson's colocalization analysis was done using the JACoP BIOP plugin in Fiji v1.54g. .
Mouse and human single cell RNA datasets were processed using Seurat (v4.1.0, https://github.com/satijalab/seurat/releases/tag/v4.1.0) within R (v4.1.1, https://github.com/r-hub/R/releases/tag/v4.1.1).
Time course analysis was performed using DEGreport (v1.30.3, https://lpantano.github.io/DEGreport/index.html).
Pathway enrichment was perfomed using EnrichR (v3.0, https://cran.r-project.org/web/packages/enrichR/).
Sample integration within human single cell RNA datasets was performed using Harmony (v0.1.0, https://github.com/immunogenomics/harmony).
Integrating external datasets into the human single cell RNA dataset was done using Seurat (v5.0.3.99911, https://github.com/satijalab/seurat/releases/tag/v5.0.3) and Azimuth (v0.5.0, https://github.com/satijalab/azimuth) within R (v4.3.1, https://github.com/r-hub/R/releases/tag/v4.3.1).
Mouse and human bulk RNA datasets were trimmed and quality filtered using FastP (v0.20.1, https://github.com/OpenGene/fastp/releases/tag/v0.20.1).
Read data was aligned using STAR (v2.7.6a, https://github.com/alexdobin/STAR/releases/tag/2.7.6a) and counted using subread-featureCounts (v2.0.1, https://subread.sourceforge.net/).
Differential expression was performed using DESeq2 (v1.28.1, https://bioconductor.org/packages/release/bioc/html/DESeq2.html) within R (v4.0.2, https://github.com/r-hub/R/releases/tag/v4.0.2).
Pathway enrichment within the bulk RNA datasets was performed using EnrichR (v3.0, above). Ligand receptor visualization leveraged NicheNetR (v1.1.0, https://github.com/saeyslab/nichenetr) and Circlize (v0.4.15, https://github.com/jokergoo/circlize) within R (v4.1.1, above).
Ggplot2 (https://github.com/tidyverse/ggplot2) and dplyr (https://github.com/tidyverse/dplyr) were leverage for figure generation and data manipulation throughout.
Mass Spectrometry phosphoprotein data was analyzed with Vanquish Neo UHPLC (Thermofisher) connected to Orbitrap Astral Mass Spectrometer (Thermofisher). global DIA raw data was processed with DIA-NN version 1.8.1 using library-free analysis mode. Protein quantification was carried out using the MaxLFQ algorithm and the number of peptides quantified in each protein group with the DiannReportGenerator Package. Phosphoproteome raw data was searched using the CHIMERYS within the Proteome Discoverer software platform v3.1 (ThermoFisher)
LC-MS data were analyzed by Compound Discover (v3.3, Thermo Scientific) and El-Maven software
Statistical analyses were carried out using Graphpad Prism Software v6.0 (Graphpad Software Inc.)

For manuscripts utilizing custom algorithms or software that are central to the research but not yet described in published literature, software must be made available to editors and reviewers. We strongly encourage code deposition in a community repository (e.g. GitHub). See the Nature Portfolio guidelines for submitting code & software for further information.

Data

Policy information about availability of data

All manuscripts must include a data availability statement. This statement should provide the following information, where applicable:

- Accession codes, unique identifiers, or web links for publicly available datasets
- A description of any restrictions on data availability
- For clinical datasets or third party data, please ensure that the statement adheres to our policy

Raw data is available under source data.

The data for this publication is available under the following accessions:
GSE226372 (human bulk RNA-Seq; https://www.ncbi.nlm.nih.gov/geo/query/acc.cgi?acc=GSE226372)
GSE227082 (mouse bulk RNA-Seq; https://www.ncbi.nlm.nih.gov/geo/query/acc.cgi?acc=GSE227082)
GSE226644 (mouse temporal scRNA-Seq; https://www.ncbi.nlm.nih.gov/geo/query/acc.cgi?acc=GSE226644)
GSE288862 (ScRNA-Seq of Human MDS and AML Bone Marrow Marrow Microenvironment; https://www.ncbi.nlm.nih.gov/geo/query/acc.cgi?acc=GSE288862).
PXD062322 (WT and TauT-/- proteomics data). The library was annotated using Mus musculus UniProt 'one protein sequence per gene' database (UP000005640_9606, downloaded April 2021).
ST003835 (WT and TauT-/- metabolomics data)
ST003836 (13C taurine tracing data).
A shiny app hosting the mouse temporal scRNA-Seq is hosted at https://wilmot-genomics.shinyapps.io/gse226644/ and was generated using ShinyCell (v2.1, https://github.com/SGDDNB/ShinyCell)

Additional Datasets:
1. Ligands were determined using NicheNet that can be accessed here:
Browaeys, R., Saelens, W. & Saeys, Y. NicheNet: modeling intercellular communication by linking ligands to target genes. Nat Methods 17, 159-162 (2020). https://doi.org:10.1038/s41592-019-0667-5
2. Cell Surface Protein atlas can be accessed here:
Bausch-Fluck, D. et al. A mass spectrometric-derived cell surface protein atlas. PLoS One 10, e0121314 (2015). https://doi.org:10.1371/journal.pone.0121314
3. AML and Healthy Immune Microenvornment Datasets can be accessed here:
Lasry, A. et al. An inflammatory state remodels the immune microenvironment and improves risk stratification in acute myeloid leukemia. Nat Cancer 4, 27-42 (2023). https://doi.org:10.1038/s43018-022-00480-0

Research involving human participants, their data, or biological material

Policy information about studies with human participants or human data. See also policy information about sex, gender (identity/presentation), and sexual orientation and race, ethnicity and racism.

Reporting on sex and gender	Studies on human samples not considered human subjects research (per NIH guidelines)
Reporting on race, ethnicity, or other socially relevant groupings	Studies on human samples not considered human subjects research (per NIH guidelines)
Population characteristics	Studies on human samples not considered human subjects research (per NIH guidelines)
Recruitment	Studies on human samples not considered human subjects research (per NIH guidelines)
Ethics oversight	Studies on human samples not considered human subjects research (per NIH guidelines)

Note that full information on the approval of the study protocol must also be provided in the manuscript.

Field-specific reporting

Please select the one below that is the best fit for your research. If you are not sure, read the appropriate sections before making your selection.

☒ Life sciences ☐ Behavioural & social sciences ☐ Ecological, evolutionary & environmental sciences

For a reference copy of the document with all sections, see nature.com/documents/nr-reporting-summary-flat.pdf

Life sciences study design

All studies must disclose on these points even when the disclosure is negative.

Sample size	No statistical method was used to predetermine sample size for experiments. We did not have any preconceived assumption regarding experiments sample size, data was collected depending on the nature of the experiments and proper statistical analysis. Adequate sample size were determined based on previous publications:<br><br>Bajaj, J., Hamilton, M., Shima, Y. et al. An in vivo genome-wide CRISPR screen identifies the RNA-binding protein Staufen2 as a key regulator of myeloid leukemia. Nat Cancer 1, 410–422 (2020). https://doi.org/10.1038/s43018-020-0054-2<br><br>Bajaj J, Konuma T, Lytle NK, Kwon HY, Ablack JN, Cantor JM, Rizzieri D, Chuah C, Oehler VG, Broome EH et al (2016) CD98-mediated adhesive signaling enables the establishment and propagation of acute myelogenous leukemia. Cancer Cell 30: 792–805<br><br>Kwon HY, Bajaj J, Ito T, Blevins A, Konuma T, Weeks J, Lytle NK, Koechlein CS, Rizzieri D, Chuah C, et al. (2015). Tetraspanin 3 Is Required for the Development and Propagation of Acute Myelogenous Leukemia. Cell Stem Cell 17, 152–164. 10.1016/j.stem.2015.06.006.
Data exclusions	No data was excluded
Replication	Each experiment was repeated 2 to 4 times as stated in the legend of each figure.
Randomization	All animals used for in vivo studies were randomly selected to receive either control or treatments. Animals were selected based on genotype. Age, Sex, and environment were controlled for. In vitro experimental samples (cell lines and primary samples) were randomly selected for control group or treatments. All other environmental factors were controlled for.
Blinding	Blinding was not relevant to this study as researchers needed to know the conditions for each experiment. Flow cytometry, FACS, western blots, seahorse, sequencing, and imaging obtain objective measurements using analytical machines therefore blinding was not necessary.

Reporting for specific materials, systems and methods

We require information from authors about some types of materials, experimental systems and methods used in many studies. Here, indicate whether each material, system or method listed is relevant to your study. If you are not sure if a list item applies to your research, read the appropriate section before selecting a response.

Materials & experimental systems

n/a	Involved in the study	
☐	☒	Antibodies
☐	☒	Eukaryotic cell lines
☒	☐	Palaeontology and archaeology
☐	☒	Animals and other organisms
☒	☐	Clinical data
☒	☐	Dual use research of concern
☒	☐	Plants

Methods

n/a	Involved in the study	
☒	☐	ChIP-seq
☐	☒	Flow cytometry
☒	☐	MRI-based neuroimaging

Antibodies

Antibodies used	A detailed list of antibodies used for flow cytometry and IF (including vendor, catalog number, and dilution) is provided in Supplementary Table 2.
Validation	All antibodies were purchased from commercial vendors and have been validated by the manufactures for use in the species and assays utilized in this study. The validation statements and published references are on the manufacturers' websites. Further in-house validation was performed with appropriate negative and positive controls for each antibody.

Eukaryotic cell lines

Policy information about cell lines and Sex and Gender in Research

Cell line source(s)	HEK293 (ATCC CRT-3216), K562 (ATCC CCL-243), THP-1(ATCC TIB-202), MV-4-11 (ATCC CRL-9591) cells were purchased from ATCC. MDS-L cells were from K. Tohyama.
Authentication	Cell lines obtained directly from ATCC were validated by vendor. MDS-L cells were authenticated in house via flow cytometry as CD45+CD34+CD38+ on 2/27/23.
Mycoplasma contamination	Cell lines were not tested for mycoplasma.
Commonly misidentified lines (See ICLAC register)	No commonly misidentified lines were used.

Animals and other research organisms

Policy information about studies involving animals; ARRIVE guidelines recommended for reporting animal research, and Sex and Gender in Research

Laboratory animals	All mice used were between 6 and 16 weeks old and both male and female mice were used.<br>Slc6a6(TauT) were maintained on the B6 background.<br>B6-CD45.1 (Strain: B6.SJL-PtprcaPepcb/BoyJ), C57BL6/J, and NSG mice (NOD.Cg-Prkdcscid Il2rgtm1Wjl/SzJ) mice were maintained as homozygous strains.<br>Cdo1fl/fl;Prrx1-Cre+ were maintained by crossing female Cdo1fl/fl with male Prrx1-Cre/+
Wild animals	The study does not involve wild animals.
Reporting on sex	Both male and female mice were used for experiments.
Field-collected samples	The study does not involve field-collected samples.
Ethics oversight	All animal experiments were performed according to protocols approved by the University of Rochester Institutional Animal Care and Use Committee. Premorbid animals were euthanised at indicated experimental time points or at end point. For all experiments, mice were monitored closely for signs of disease or morbidity daily and were sacrificed for visible signs of hunched dorsum, failure to thrive, or any signs of infection. These limits were not exceeded for any experiment.

Note that full information on the approval of the study protocol must also be provided in the manuscript.

Flow Cytometry

Plots

Confirm that:

☒ The axis labels state the marker and fluorochrome used (e.g. CD4-FITC).

☒ The axis scales are clearly visible. Include numbers along axes only for bottom left plot of group (a 'group' is an analysis of identical markers).

☒ All plots are contour plots with outliers or pseudocolor plots.

☒ A numerical value for number of cells or percentage (with statistics) is provided.

Methodology

Sample preparation	For KLS, bone marrow was recovered from femurs, tibias, and pelvis. Bones were crushed using a mortar and pestle, cells were suspended in Hanks' balanced salt solution (HBSS) (Gibco, Life Technologies) containing 5% (vol/vol) fetal bovine serum and 2 mM EDTA and filtered using a 70um filter. Red blood cells were lysed using RBC Lysis Buffer (eBioscience) and ckit cells were enriched by positive selection using magnetic beads (Miltenyi Biotec) on a AutoMACS Pro (Miltenyi Biotec) before staining for lineage markers. All antibodies were purchased from BD Pharmingen, eBioscience or BioLegend.<br><br>Analysis of leukaemic mice was similarly done. Briefly, cells, spleens and/or bone marrow from sick mice were prepared as described above to generate single cell suspensions that were then incubated with FACS antibodies.<br><br>Mesenchymal stromal cells (MSCs) were isolated from leukaemic mice and cultured in 10cm dishes in MEM α with no ascorbic acid (Gibco) supplemented with 15% FBS and 100 IU/mL Penicillin/Streptomycin (Gibco). 6 days post culture initiation, the cells were sorted for MSCs.<br><br>For BM stromal cells, bone and bone marrow (BM) were isolated from long bones and pelvis in 1x Media 199 (Gibco) with 2% fetal bovine serum (GeminiBio). BM was digested for 30 minutes in HBSS containing 2mg/mL Dispase II (Gibco), 1mg/mL Collagenase Type IV (Sigma-Aldrich), and 20ng/mL DNase Type II (Sigma-Aldrich). Bone spicules were digested for 60 minutes in PBS supplemented with 2.5mg/mL Collagenase Type I (Stem Cell Technologies) and 20% FBS. Digested bone marrow was RBC lysed using RBC Lysis Buffer (eBioscience). Bone and BM cells were pooled and CD45+ Ter119+ hematopoietic cells were magnetically depleted on an autoMACS cell separator (Miltenyi Biotec). The CD45-Ter119- stromal cells were either stained an analyzed for candidate populations by flow cytometry (BD LSRFortessa) or further enriched by sorting (BD FACSAria II) and processed for single cell RNA-Sequencing.<br><br>Lineage depleted TauT+/+ and TauT-/- murine leukeamia cells were fixed using BD Cytofix/Cytoperm Fixation/ Permeabilization Kit (BD Bioscience) per manufacturer's protocols. Cells were stained with primary antibody against phospho-mTOR (Cell Signaling Technologies). Cells were then stained with donkey anti-rabbit secondary antibody conjugated with Alexa Fluro 488 (Invitrogen) to detect mTOR. Analysis was performed on LSRFortessa (Becton Dickinson). Data was analyzed using FlowJo software.
Instrument	Analysis was carried out by LSRFortessa and cell sorting was carried out on a FACSAria II (BD Biosciences).
Software	FACSDiva v9.0 (BD Biosciences) was used to collect data and data analysis was done using FlowJo v10.9.0. (Tree Star Inc.).
Cell population abundance	The purity of post-sort KLS from TauT +/+ and TauT-/- mice was higher than 90% as determined by flow cytometry.<br>The purity of post-sort transduced KLS cells was greater that 95% as determined by flow cytometry.
Gating strategy	KLS sort: morphology (FSC/SSC) -> live cells (PI-) -> lineage negative (lin-) -> ckit + & Sca1 + (ckit/Sca1).<br>BCR-ABL & NUP98-HOXA9 transduced KLS: morphology (FSC/SSC) -> live cells (PI-) -> NGFR+ (BCR-ABL vector) & CD2+ (NUP98-HOXA9 vector).<br>Primary and Secondary bcCML Sort: morphology (FSC/SSC) -> live cells (PI-) -> NGFR+/GFP (BCR-ABL vector) & CD2+/YFP (NUP98-HOXA9 vector) -> lineage negative (lin-).<br>Secondary AML Sort: morphology (FSC/SSC) -> live cells (PI-) -> NGFR+ (MLL-AF9 vector) & YFP (NRAS vector) -> Ckit positive (Ckit+).<br>shRNA transduced bcCML and human samples: morphology (FSC/SSC) -> live cells (PI-) -> GFP+ (shRNA vector).<br>Stromal cell Sort/analysis: morphology (FSC/SSC) -> live cells (PI-) -> GFP- (Cancer Negative) ->CD45-/Ter119- -> CD31-(endothelial) -> CD51+(osteo) -> Sca-1+ or CD140a<br>MSC sort/analysis: morphology (FSC/SSC) -> live cells (PI-) ->CD45-/Ter119 -> CD31-(endothelial) -> CD51+(osteo) -> Sca-1+<br>Flow cytometry-based pMTOR expression: morphology (FSC/SSC) -> mCherry/RagA+ (if infected) -> pMtor+<br>Boundaries between "positive" and "negative" populations were defined using a fluorescence minus one or unstained samples.<br>See Supplementary Table 2 for antibody panels used.

☒ Tick this box to confirm that a figure exemplifying the gating strategy is provided in the Supplementary Information.
